# An Efficient High Throughput Metabotyping Platform for Screening of Biomass Willows

**DOI:** 10.3390/metabo4040946

**Published:** 2014-10-28

**Authors:** Delia I. Corol, Claudia Harflett, Michael H. Beale, Jane L. Ward

**Affiliations:** Department of Plant Biology and Crop Sciences, Rothamsted Research, West Common, Harpenden, Herts AL5 2JQ, UK; E-Mails: delia.corol@rothamsted.ac.uk (D.I.C); claudia.harflett@rothamsted.ac.uk (C.H.); mike.beale@rothamsted.ac.uk (M.H.B.)

**Keywords:** willow, *Salix*, metabolomics, metabotyping, metabolite-QTL, NMR

## Abstract

Future improvement of woody biomass crops such as willow and poplar relies on our ability to select for metabolic traits that sequester more atmospheric carbon into biomass, or into useful products to replace petrochemical streams. We describe the development of metabotyping screens for willow, using combined 1D ^1^H-NMR-MS. A protocol was developed to overcome 1D ^1^H-NMR spectral alignment problems caused by variable pH and peak broadening arising from high organic acid levels and metal cations. The outcome was a robust method to allow direct statistical comparison of profiles arising from source (leaf) and sink (stem) tissues allowing data to be normalised to a constant weight of the soluble metabolome. We also describe the analysis of two willow biomass varieties, demonstrating how fingerprints from 1D ^1^H-NMR-MS vary from the top to the bottom of the plant. Automated extraction of quantitative data of 56 primary and secondary metabolites from 1D ^1^H-NMR spectra was realised by the construction and application of a *Salix* metabolite spectral library using the Chenomx software suite. The optimised metabotyping screen in conjunction with automated quantitation will enable high-throughput screening of genetic collections. It also provides genotype and tissue specific data for future modelling of carbon flow in metabolic networks.

## 1. Introduction

Short rotation coppice (SRC) willow (*Salix spp*.) is an established biomass crop that is currently used as a feedstock for heat and power generation, and has potential for future production of biofuels and other industrial products. Genetic improvement of SRC-willow has been carried out by conventional plant breeding techniques and this has led to new commercial varieties, selected for increased pest resistance and biomass yield [1]. To develop further the potential of this crop, a molecular genetic approach to identifying key genes is being used to accelerate the improvement process via marker assisted breeding [2], as demonstrated by a recent report on quantitative trait mapping of loci (QTL mapping) for pathogen resistance [3]. To underpin this endeavour Rothamsted Research maintains an extensive *Salix* germplasm bank, including some 1500 accessions in the National Willow Collection gathered from around the globe, and a significant number of mapping populations, some which contain almost 1000 progeny. A high resolution willow genetic map, aligned with that of the related poplar (for which a full genome sequence is available), has been established [4], as have extensive agronomic trials in a variety of nutrient and water supply situations.

Many of the quality traits that are targets for willow improvement e.g., biomass yield, calorific value, pest resistance and value-added chemicals are intimately linked with the operation of the plant metabolic network, as it responds to genetic and environmental programming. QTL-mapping of metabolite levels (mQTL analysis) will lead to biochemical pathways and genes that can be associated with desirable traits [5,6,7]. To develop the mQTL approach, methods for screening the extensive genetic collections are a necessity and plant metabolomics technology has developed to an extent where such large-scale screens are possible. Metabolomics analysis usually involves the application of 1 dimensional proton nuclear magnetic resonance (1D ^1^H-NMR) spectroscopy and mass spectrometry (MS) in a combination of unbiased “metabolite fingerprinting” of un-purified solvent extracts, with more targeted quantitative analysis of known compounds [8,9]. In metabolite fingerprinting, the use of chemometrics to mine datasets for “metabolite biomarkers”, and correlative statistics to relate metabolite features to genetic markers are now established technologies [5,6,10,11]. Key factors in generating high quality data in large scale metabolomic fingerprinting experiments are experimental design, sampling and sample stability. This leads to spectral stability which is absolutely required for confidence in data mining.

1D ^1^H-NMR is routinely used in plant metabolomics due to its high spectral reproducibility and low instrument drift [12]. However this relies on plant extracts that are comparable such that all peaks appear in consistent positions along the chemical shift scale and that peak resolution between samples is equivalent. Factors that impact on spectral quality and comparability between samples includes pH variation, differences in ionic strength and peak broadening due to the presence of paramagnetic and other metal cations [13,14,15]. These problems impact differentially on resonances from different compound classes and often need to be addressed prior to data collection. The use of buffered NMR solvents to normalise pH across samples is regularly used in plant metabolomics to align peaks [14,15,16,17], although as an alternative, new software algorithms exist to adjust for pH variation [18,19]. Complexation with chelators such as ethylenediaminetetraacetic acid (EDTA) addresses peak broadening from the presence of metal cations [14,17,20]. In extreme cases, peak broadening is highly variable across datasets and even can lead to apparent loss of peaks into the spectrum baseline.

Hence, the development of robust protocols for sample handling and data collection are essential components of any mQTL screen, where many hundreds of samples are involved. Willow (and other tree species) present a range of problems to large-scale screening and metabolomics data collection, which has been established on more tractable species such as Arabidopsis [21,22,23], with other significant studies on Solanaceae [24,25], cereals [26,27] and Medicago [28]. Metabolite screening of perennial woody plants has been reported for loblolly pine (for milled stem tissue) [29], but generally the heterogeneity of tissue types and physical/chemical properties requires considerable re-thinking of the protocols developed for annual crops. In this paper we describe the development of new protocols that allow stable 1D ^1^H-NMR and MS data collection on both leaf and stem tissue of SRC willow. The utility and robustness of the method is demonstrated in a study of source and sink metabolites in two willow biomass genotypes. We have also further developed the method for high throughput genetic screens, including automated quantitation using a bespoke 1D ^1^H-NMR spectral library.

## 2. Results and Discussion

### 2.1. Establishment of a Robust 1D ^1^H-NMR-MS Protocol for Willow Metabolite Screening

We had established a number of years ago that 1D ^1^H-NMR profiling of extracts of freeze-dried Arabidopsis aerial tissue, made directly into deuterated methanol-water mixtures produced stable spectral fingerprints containing a range of primary and secondary metabolites that could define different genotypes [21,30,31]. When this method was applied to wheat flour, a small modification, to incorporate a brief 2 min/90 °C heat shock, was added to the protocol in order to denature hydrolytic enzymes that remained active in the NMR samples causing spectral instability, particularly in carbohydrate signatures [26]. This modified procedure has since been applied to over 100,000 samples of leaf, stem and seed tissues in our laboratory over recent years and has been described in detail [32,33]. The utility of this method is further enhanced as aliquots of the extract can be taken and diluted with non-deuterated solvent to provide parallel samples for mass fingerprinting by electrospray ionisation mass spectrometry (ESI-MS). These samples are totally compatible with the electrospray technique and can be infused directly into spectrometers and/or subjected to full LC-MS analysis. As the identical samples are used, correlative statistical analysis of 1D ^1^H-NMR *versus* ESI-MS datasets has credibility and adds much confidence to biomarker discovery and structural determination (for example [34]).

**Figure 1:**
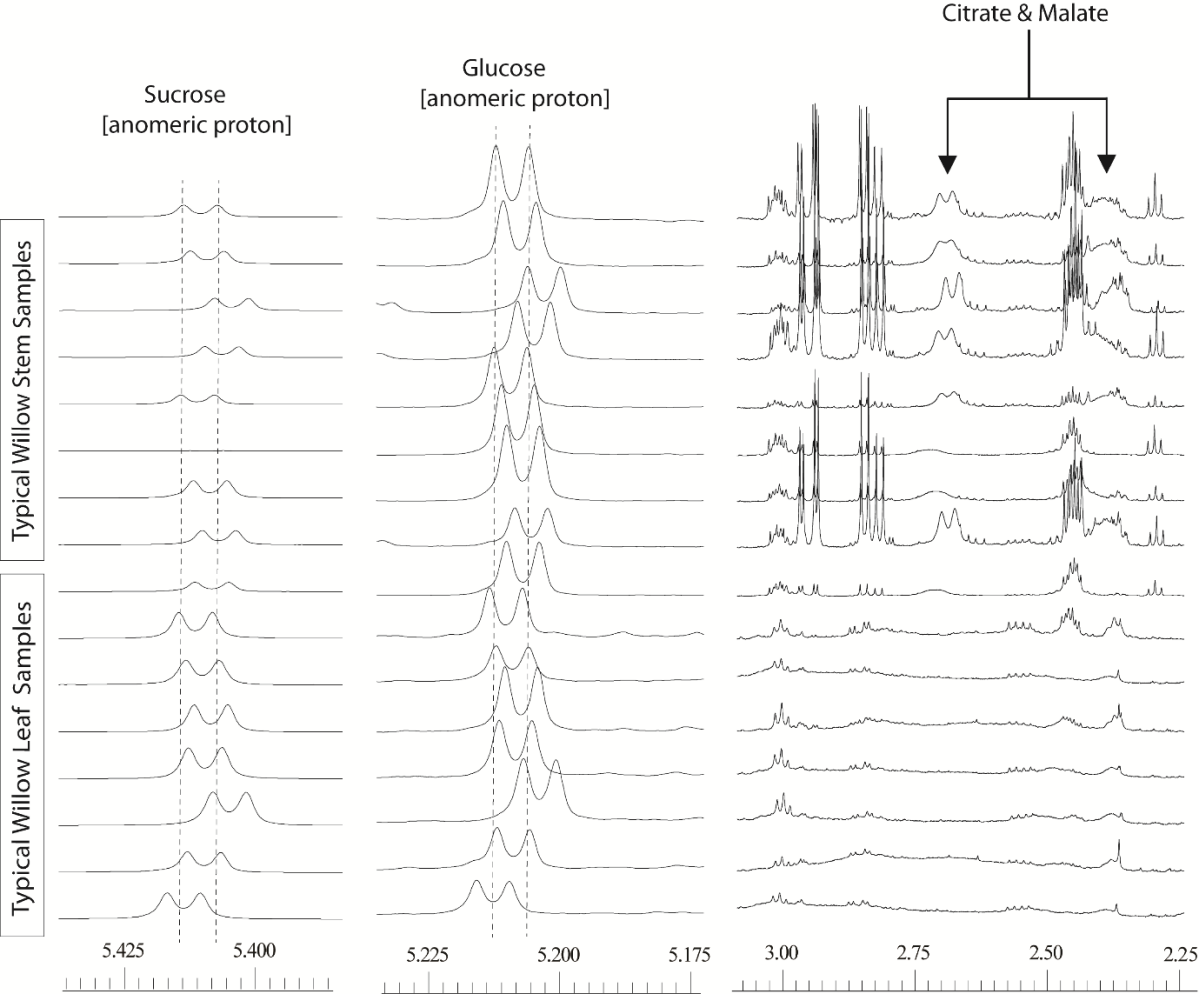
600 MHz 1D ^1^H-NMR willow leaf and stem spectra, from a polar solvent extraction using 80:20 D_2_O:CD_3_OD: illustrating chemical shift variation in anomeric sucrose (δ5.425–5.400) and glucose (δ5.225–5.195) signals together with shift variation and broadness in citrate and malate signals (δ2.75–2.30).

In initial experiments with willow, we utilised freeze-dried leaf and stem tissue, taken from three parts (top, middle, bottom) of the two biomass varieties, Tora and Resolution. Plant tissue was harvested, from field plots, in June in the middle of the rapid growth season, after coppicing in the previous February. It soon became apparent that 1D ^1^H-NMR fingerprints generated by our standard protocol (extraction at 50 °C in 80:20 D_2_O:CD_3_OD) [32,33] suffered from two problems: some peaks were poorly resolved and secondly many signals (compounds) common to all tissues were misaligned relative to added d_4_-3-(trimethylsilyl)propionic acid (d_4_-TSP) internal calibration standard (Figure 1). The degree to which these two problems manifested themselves varied across the dataset. Misalignment of peaks was not a simple linear shift that could easily be dealt with by adding a data processing step. Binning or “bucketing” the 1D ^1^H-NMR spectra is a technique which is commonly utilised in metabolomics prior to downstream processing with statistical software. The technique reduces the resolution of the dataset to ensure that small changes in chemical shift between spectra do not yield false results from statistical processing of the data. The width (in ppm) of the “bucket” is chosen to try and ensure that a peak remains in its given bin or “bucket” despite small chemical shift variations between analyses. This can be achieved by using a user-defined fixed bucket width or via the use of intelligent bucketing [35] which uses an algorithm to set the optimum bucket width for particular peaks such that they are not split between buckets. However, the extent of the variation in chemical shift for the distinctive anomeric hydrogen signals of sucrose and α-glucose (Figure 1) was such that application of normal data processing strategies resulted in these abundant metabolites residing is different spectral buckets (bins).

A fix based on processing with very wide bins (either via manual definition of the bucket size, or via intelligent bucketing) to encompass these shifts was not feasible as this resulted in signals from normally separated metabolites falling into the same bin, effectively reducing the high resolution spectra to a less useful, low resolution dataset with many uncertainties in metabolite annotation. The separate problem of poor resolution was also evident for a number of spectral regions particularly for the malate and citrate signals. In stem tissue samples, these signals could be easily observed but the degree of peak broadness varied for one sample to another depending on the harvest point of the willow stem. In leaves, the signals were so broad that they often seemingly disappeared into the baseline. The dual problem of variable line width and poor alignment meant that samples from different tissues or those taken from different parts of the plant could not easily be compared.

A similar problem has previously been observed in extracts of fruit tissue such as tomato and fruit juices [36,37] that contain varying levels of malic and citric acids. In fruit juices, the problem was easily rectified by adding buffer directly to the liquid sample. In tomato tissues the problem was overcome by modifying the protocol to add a dry-down step after the initial extraction and removal of aliquots for ESI-MS, followed by re-dissolution of the NMR sample in deuterated phosphate buffer. This stabilised the 1D ^1^H-NMR line shape and chemical shift of the organic acids as described by Kim *et al.* [38] and also realigned slight pH shifts in distinctive carbohydrate anomeric hydrogens. The willow spectra revealed that this plant also has high levels of citric and malic acids, but unfortunately, the relatively straight forward dry down/buffering solution to the problem was not completely successful (Table 1). It is known that willow is unusual in that it accumulates high levels of calcium oxalate in leaf tissue [39] and we reasoned that the 1D ^1^H-NMR alignment problems were due to complex interactions of calcium ions with a variety of organic acids in the matrix, including malate and citrate as well as the 1D ^1^H-NMR-invisible oxalate. To investigate this problem we carried out a detailed array of experiments as shown in Table 1, involving buffering at different pHs and ionic strengths and the addition of variable amounts of EDTA to complex the calcium ions. Initial trials were carried out on a dried down polar extract (80:20 H_2_O:CH_3_OH) of plant tissue. Reconstitution in 300 mM sodium phosphate buffer at pH6 failed to align the 1D ^1^H-NMR peaks or to sharpen poorly resolved peaks such as those of citrate and malate. Increasing the ionic strength of the buffer to 600 mM still did not improve resolution. Trials were then carried out using EDTA to complex the Ca^2+^ in the sample (Table 1). Addition of 10 µL of a 3.2 mM solution of EDTA began to sharpen the pair of citrate doublets which appear between δ2.50 and 2.75. However the position of these peaks varied between samples. Adding increasing amounts (up to 100 µL) of the 3.2 mM solution of EDTA sharpened these peaks further but did not completely stabilise the chemical shift. Alternate strategies, to deal with Ca^2+^, such as precipitation as CaF_2_ following potassium fluoride addition [40] or removal by chelation with solid cation exchange resins [41] were also unsuccessful, failing to improve resolution or stability of peak position.

An alternate solution to re-dissolution of the dried extract in aqueous buffer was to reconstitute the sample in the same ratios of deuterated methanol-water solvents as used to extract the plant. This improved the efficiency of reconstitution. Buffering of this solution via the addition of a small concentrated (10 µL, 2.6 M) “slug” of pH 6.0 buffer to the final sample appeared to improve the alignment of most signals in the spectrum, excluding malate and citrate. Increase of the pH of the concentrated buffer additive to 7.4 or 8.0 resulted in good alignment of these signals. Sharpening of the citrate and malate signals, such that they were of a comparable resolution across different tissues and genotypes, also required the addition of EDTA and after further experimentation it was found that a 10 µL addition of a stronger solution (32 mM) worked most effectively. The addition of this EDTA solution however, required further adjustments to buffer concentration to re-align some signals. It was found that the addition of a further 10 µL portion of the 2.6 M buffer such that the final solution was supplemented with 10 µL 32 mM EDTA and 20 µL 2.6 M potassium phosphate (pH 7.4) was optimum.

**Table 1:** Matrix of methods attempted to align and sharpen willow 1D ^1^H-NMR signals.

Initial Extraction Solvent ^†^	Dry Down Step	Reconstitution Solvent ^†^ (pH/Ionic Strength)	Additives ^‡^		Spectral Quality		
			Additive	Final Concentration in NMR Tube	Peak Resolution (Citrate & Malate)	Peak Alignment (Citrate & Malate)	Peak Alignment (Other Peaks)
A	Yes	C (6.0/300mM)	None	N/A	Poor	Poor	No
A	Yes	C (6.0/600mM)	None	N/A	Poor	Good	No
A	Yes	C (6.0/600mM)	10 µL 3.2 mM EDTA (D_2_O)	45 μM	Good	Poor	Aligned within, but not across, tissues
A	Yes	C (6.0/600mM)	30 µL 3.2 mM EDTA (D_2_O)	131 μM	Good	Poor	Aligned within, but not across, tissues
A	Yes	C (6.0/600mM)	50 µL 3.2 mM EDTA (D_2_O)	213 μM	Good	Poor	Aligned within, but not across, tissues
A	Yes	C (6.0/300mM)	Cation exchange resin (Chelex 100, Na form) *	N/A	Poor	Poor	No
A	Yes	C (6.0/300mM)	10 µL 2M KF (H_2_O)	28 mM	Poor	Poor	Aligned within, but not across, tissues
A	Yes	C (7.0/300mM)	None	N/A	Poor	Poor	No
A	Yes	C (7.0/300mM)	50 µL 3.2 mM EDTA (D_2_O)	213 μM	Good	Poor	No
A	Yes	C (6.0/300mM)	100 µL 3.2 mM EDTA (D_2_O)	400 μM	Variable	Poor	No
B	No	N/A	100 µL 3.2 mM EDTA (D_2_O)	400 μM	Poor	Poor	Yes
B	No	N/A	10 µL 32 mM EDTA (D_2_O)	450 μM	Poor	Poor	No
A	Yes	B	10 µL–2.6 M Potassium Phosphate Buffer (D_2_O), **pH = 7.4**	37 mM	Poor	Good	Yes
A	Yes	B	10 µL–2.6 M Potassium Phosphate buffer (D_2_O), **pH = 7.4**; 10 µL–32 mM EDTA (D_2_O)	36 mM (Pi) 444 μM (EDTA)	Good	Poor	Yes
A	Yes	B	10 µL–2.6 M Potassium Phosphate buffer (D_2_O), **pH = 8.0**; 10 µL–32 mM EDTA (D_2_O)	36 mM (Pi) 444 μM (EDTA)	Good	Good	Yes
A	Yes	B	20 µL–2.6 M Potassium Phosphate buffer (D_2_O), **pH = 8.0**; 10 µL–32 mM EDTA (D_2_O)	71 mM (Pi) 438 μM (EDTA)	Good	Poor	Yes
A	Yes	B	20 µL–2.6 M Potassium Phosphate buffer (D_2_O), **pH = 8.0**; 20 µL–32 mM EDTA (D_2_O)	70 mM (Pi) 865 μM (EDTA)	Good	Poor	Yes
A	Yes	B	20 µL–2.6 M Potassium Phosphate buffer (D_2_O), **pH = 7.4**; 10 µL–32mM EDTA (D_2_O)	71 mM (Pi) 438 μM (EDTA)	Good	Excellent (within a 0.01 ppm bin width)	Yes
B	No	N/A	20 µL–2.6 M Potassium Phosphate buffer (D_2_O), **pH = 7.4**; 10 µL–32 mM EDTA (D_2_O)	71 mM (Pi) 438 μM (EDTA)	Good	Excellent (within a 0.01 ppm bin width)	Yes

**† Solvents: A** = H_2_O:CH_3_OH (4:1) (1mL); **B** = D_2_O:CD_3_OD (4:1), containing 0.01% d_4_-TSP (1 mL); **C** = Sodium phosphate in D_2_O, containing 0.05% d_4_-TSP (750 µL). ^‡^ Additions are made to final NMR aliquot (700 µL) from which 650 μL was removed for spectrum collection; * Solid resin was added to the reconstituted extract in buffer, and incubated for 20 min before supernatant (650 μL) was removed for spectrum collection.

**Figure 2:**
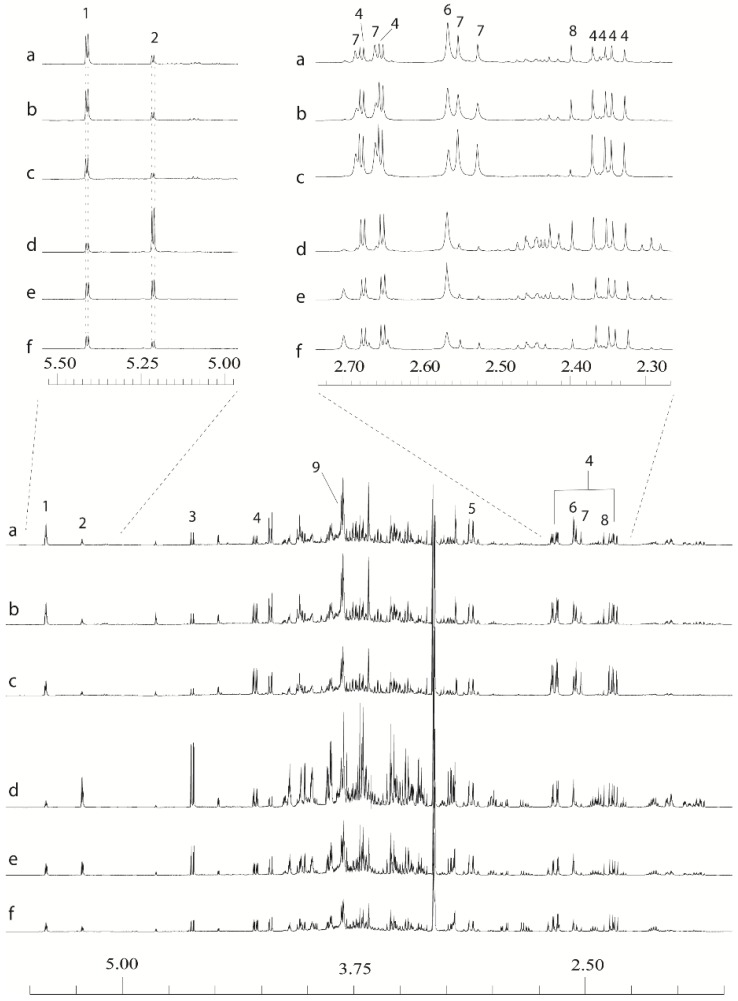
Examples of leaf and stem 1D ^1^H-NMR data derived from extracts made using an:80:20 D_2_O:CD_3_OD extraction with final additions of 20 µL 2.6 M potassium phosphate buffer and 10 µL 32 mM ethylenediaminetetraacetic acid (EDTA) solutions in D_2_O. (**a**) Resolution-leaf-top; (**b**) Tora-leaf-middle; (**c**) Resolution-leaf-bottom; (**d**) Tora-stem-top; (**e**) Resolution-stem-middle; (**f**) Resolution-stem-bottom. 1: sucrose; 2: α-glucose; 3: β-glucose; 4: malate; 5: Ca-EDTA^2−^; 6: Ca-EDTA^2−^; 7: citrate; 8: succinate; 9: free EDTA.

In this way, a dataset was achieved within which all peaks from all tissue types were well resolved and aligned such that bucketing to 0.015 ppm reliably captured all the peaks in the same buckets between samples. By this approach we developed a protocol that produced stable, reproducible 1D ^1^H-NMR spectra whilst retaining the ability to remove aliquots of the original extract for ESI-MS. To prevent introduction of EDTA and buffer salts into ESI-MS samples, concentrated chelator and buffer solutions were added at the end of the process only to the NMR sample. Representative spectra from stem and leaf tissues are shown in Figure 2. It can be seen that the organic acids are now well resolved and aligned, as are the anomeric hydrogens from common sugars. The signals from the Ca^2+^ complex of EDTA are visible at 3.1 ppm (quartet) and 2.55 (singlet) [42,43] as abundant peaks, but do not interfere with those from endogenous metabolites. We can’t rule out the possibility that EDTA was also complexing with other paramagnetic and diamagnetic metal ions but characteristic 1D ^1^H-NMR peaks for e.g., Mg-EDTA (2.8 ppm) [42] or Mn-EDTA (2.8 ppm) [20] were not seen suggesting that Ca^2+^ was the major cation responsible for chemical shift variation and peak broadening in willow tissues. Diamagnetic cations such as Ca^2+^, are commonly associated with chemical shift variation due to their ability to bind to metabolites such as citrate [40]. However, it is unusual for these diamagnetic cations to affect peak resolution which normally arises due to paramagnetic ion content. For example, studies in saliva showed that no peak broadening of the citrate peaks occurred due to the addition of additional Ca^2+^ [44]. In willow tissues it appears that the variable organic acid content in leaf and stem tissues coupled with a high calcium oxalate presence, especially in leaves is influencing not just peak position but also resolution of both malate and citrate peaks, a situation that varies with the age of the tissue and which cannot be rectified by buffering alone, instead requiring a careful balance of metal chelator addition and pH adjustment.

As the newly developed method involved a dry-down step, it also presented an opportunity to record the mass of extracted metabolites from each of the different tissue types. As shown in Table 2 the total mass of metabolites extracted from standard aliquots of freeze-dried milled willow tissue varied with the location of sampling.

**Table 2:** Level of extractable metabolite pool from *S. viminalis* leaf and stem tissue, expressed as a % of total dry biomass.

Tissue and Position	Tora % Extractable	Resolution % Extractable
Leaf–Top	26.9 ± 1.7	26.4 ± 2.6
Leaf–Middle	28.2 ± 1.7	31.6 ± 3.4
Leaf–Bottom	31.1 ± 1.2	30.0 ± 3.7
Stem–Top	32.00 ± 2.9	32.4 ± 2.3
Stem–Middle	18.3 ± 1.8	18.1 ± 1.8
Stem–Bottom	11.9 ± 1.7	13.8 ± 1.9

On the whole, approximately 30% of the dry mass of willow leaf was extractable, and this was consistent across both older and younger leaves. However, for stem tissue, not surprisingly, the percentage of extractable metabolites per unit dry weight of tissue, decreased from *ca*. 32% in stem tissue taken from the top of the plant to just 12% in stem material harvested from the bottom of the plant, reflecting the maturity and hardness of the wood from top to bottom. For qualitative analysis and relative quantitative analysis *i.e.*, within sample or across samples of the same tissue type, the lower amount of extractives is not an issue. However, for the calculation of carbon pools and flow in different tissues around the plant then the extractable mass becomes a factor in any mass-balance analysis.

**Figure 3:**
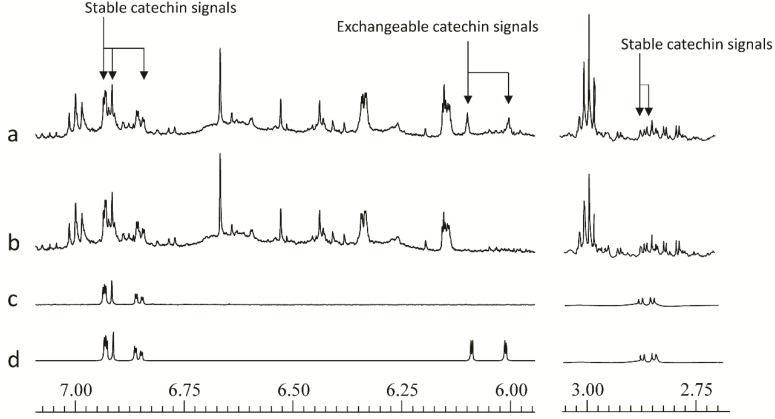
600 MHz 1D ^1^H-NMR spectral regions from δ7.075–5.95 and δ3.05–2.70 to illustrate the position of stable and deuterium-exchangeable catechin signals of (**a**) freshly extracted willow leaf extract, (**b**) 12 h old willow leaf extract, (**c**) 12 h old catechin standard, (**d**) freshly extracted catechin standard.

A further issue that came to light during the development of the method concerns the flavan-3-ol catechin, which occurs widely in the plant kingdom, and is present at significant levels in willow samples. On standing in buffered deuterated aqueous solvents this compound undergoes slow hydrogen-deuterium exchange at the C-6 and C-8-positions.This results in loss of signal at δ6.09 (H-6) and δ 6.00 (H-8). Although less rapid than hydroxyl or carboxyl hydrogen exchange, the exchange of these aromatic hydrogen atoms, via keto-enol tautomerism, was a fairly fast process and as shown in Figure 3, and was complete in 12 h at pH 7.4. The phenomena of H/D exchange have previously been reported in response to heating samples containing flavonoid metabolites [45,46] and also in related anthocyanin molecules in acidified methanolic or aqueous solutions [47]. For the operation of the high throughput screen, varying degrees of exchange of the catechin H-6 and H-8 hydrogens, have potential to give false positive results in multivariate analyses of large sets of spectra. This can be avoided by either “resting” the samples for 12 h after addition of the buffer solution, before data collection, or, by removal of the affected chemical shift “bins” from the spreadsheet of chemical shift *versus* intensity during data processing [32]. This will prevent false discovery of catechin as a biomarker. Other non-exchangeable catechin aromatic hydrogens at δ6.93, 6.92 and 6.85, together with the aliphatic double doublet at δ2.86 (Figure 3) can be diagnostic for this compound and thus should emerge from multivariate analysis if levels are changing across a sample set It should be noted that hydrogen-deuterium exchange in flavonoids only affects the buffered NMR sample. Samples for ESI-MS were removed before re-dissolution in NMR solvent and thus the flavonoids do not undergo any molecular weight shifts in this screen.

### 2.2. Analysis of Tora and Resolution Using the New Method

Willow stems and leaves from the two biomass varieties Tora and Resolution were analysed using the protocol described above. The choice to analyse two biomass willow varieties which are genetically related was deliberately made in order to test the robustness of the newly developed extraction and data collection protocol. Unlike many other biomass willows, these two varieties have a very similar phenotype and metabolite changes due to genotype were expected to be subtle. The ability of a protocol to separate spectra arising from these genotypes relied on high quality analytical data with a low variation due to the method itself. Average relative standard deviations, describing variation in technical replication, for abundant metabolites identified in the leaf and stem 1D ^1^H-NMR spectra ranged from 2%–8% (Table 3).

**Figure 4:**
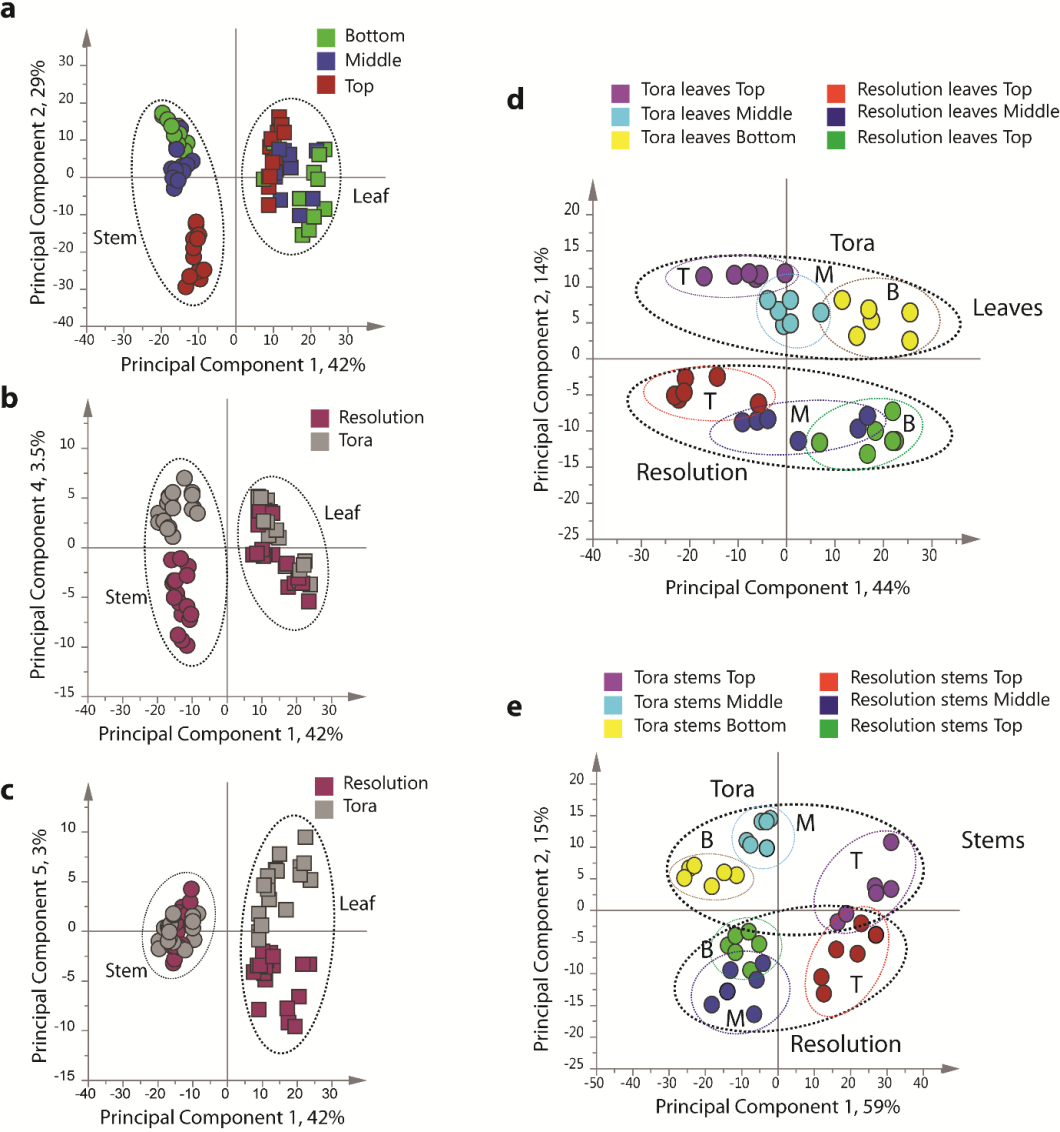
PCA scores plots of binned 1D ^1^H-NMR data, indicating clustering of Tora and Resolution leaf and stem samples. (**a**) PC1 *vs.* PC2 of leaf and stem data, coloured by harvest position; (**b**) PC1 *vs.* PC4 of leaf and stem data, coloured by genotype; (**c**) PC1 *vs.* PC5 of leaf and stem data, coloured by genotype; (**d**) PC1 *vs.* PC2 of leaf data only, coloured by genotype and harvest position; (**e**) PC1 *vs.* PC2 of stem data only, coloured by genotype and harvest position. Harvest position: B:bottom; M:middle; T:top.

PCA of the resultant full 1D ^1^H-NMR dataset (Figure 4), including all replicates, showed good clustering of the experimental data. Samples from technical and biological replicates for relevant samples clustered together and showed a lower variance compared to material from different sampling position or that from differing genotypes. Unsurprisingly the largest separation within the PCA model, in the direction of PC1 accounting for 42% of the total variance, was observed between leaf and stem samples (Figure 4a) irrespective of genotype or sampling point. PC2, accounting for 29% of the variance, described the separation within the leaf or stem cluster, due to sampling point (top, middle or bottom of the plant). The impact of sampling point was greatest in stem samples where samples harvested from the top of the plant formed a distinct cluster. When coloured according to genotype, PC4, which accounted for 3.5% of the total variance, separated the two biomass lines in the stem samples (Figure 4b).

**Table 3:** Relative standard deviations (RSD) observed for characteristic metabolite regions in leaf and stem 1D ^1^H-NMR data. Data is based on three technical replicates per biological sample. Reported values represent the average % RSD observed across all leaf or stem samples. n.d. denotes a metabolite that was not quantified in a particular tissue.

Metabolite	Leaves % RSD	Stems % RSD	Metabolite	Leaves % RSD	Stems % RSD
Sucrose	2	3	GABA	8	4
Glucose	4	3	Glutamine	2	2
Fructose	2	2	Alanine	3	2
Myo-inositol	2	n.d.	Threonine	4	3
Succinate	6	7	Valine	5	5
Citrate	3	3	Isoleucine	6	4
Malate	2	2	Leucine	4	3
Ascorbate	4	7	2-Phenylethylamine	5	4
Quinate	4	3	Catechin	2	7
Lactate	n.d.	3	Dihydromyricetin	3	8
Aspartate	4	8	Gallocatechin	3	8
Asparagine	7	4	Chlorogenic Acid	3	n.d.

In leaf samples, the two genotypes could be separated by PC5 accounting for 3% of the total model variance (Figure 4c). When leaf and stem samples were analysed separately (Figure 4d,e), clear clusters could be seen for sampling point in the direction of PC1 in both models. Separation due to genotype was evident in PC2. Interestingly, in stem tissue, the greater discrimination of samples was observed for tissues harvested from the bottom or middle of the plant. This discrimination was less evident in leaf samples where genotypes could be separated at all positional harvest points. Technical replication could also be assessed in the models resulting from separate tissue types (Figure S1) and in general variance between the three technical replicates was lower than that observed between biological replicates.

**Figure 5:**
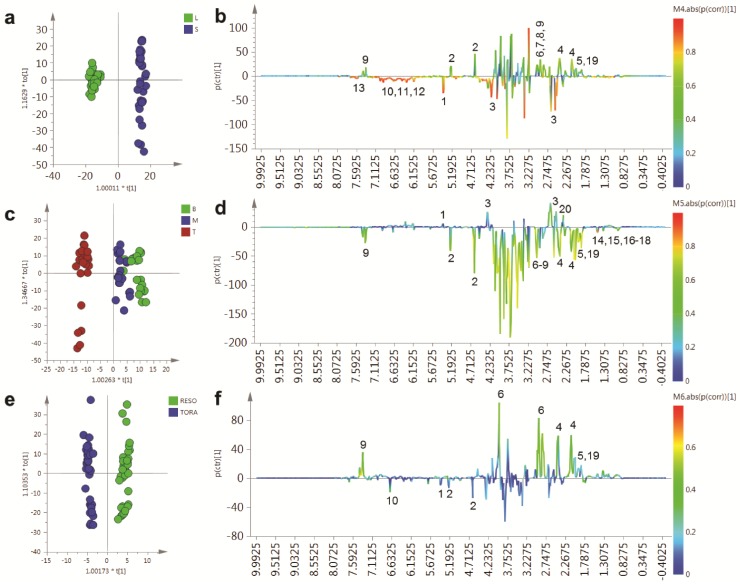
OPLS analysis of binned 1D ^1^H-NMR data. (**a**) OPLS scores plot with Y variable as tissue type; (**b**) OPLS S-Line plot describing differences between stem (positive) and leaf (negative); (**c**) OPLS scores plot with Y variable as harvesting position; (**d**) OPLS S-Line plot describing differences between tissue harvested from the bottom of the plant (positive) and the top of the plant (negative); (**e**) OPLS scores plot with Y variable as genotype; (**f**) OPLS S-Line plot describing differences between Resolution (positive) and Tora (negative); Peak IDs: 1: sucrose; 2: glucose; 3: malate; 4: glutamine; 5: glutamate; 6: asparagine; 7: aspartate; 8: GABA; 9: 2-phenylethylamine; 10: dihydromyricetin; 11: catechin; 12: gallocatechin; 13: chlorogenic acid; 14: alanine; 15: threonine; 16: leucine; 17: isoleucine; 18: valine; 19: quinate; 20: citrate.

In order to determine the metabolites responsible for these distinct separations, a series of O-PLS models were constructed using a dummy matrix for separations due to tissue, sampling point or genotype (Figure 5). Differences in the abundant metabolites between stems and leaves are shown in the OPLS S-plot in Figure 5b. Stem tissues typically contain higher glucose than leaves. In addition a number of amino acids are elevated including glutamine, asparagine, aspartate and GABA. The aromatic metabolite 2-phenylethylamine, a metabolite formed from phenylalanine and which is dominant in juvenile willow tissues is more abundant in stem tissues. Finally, signals relating to quinic acid at δ 1.845–2.073 are present in both tissues but are elevated in stem tissues and are also discriminatory metabolites.

Contrastingly, leaf samples contain higher sucrose levels and elevated amounts of the organic acid malate. The abundant secondary metabolites, observed in leaves, included catechin and gallocatechin, while dihydromyricetin, the most abundant flavonoid in these *Salix* genotypes, was higher in leaves compared to stem samples. Finally, chlorogenic acid, an ester formed from caffeic and quinic acids was detected only in leaf samples. Figure 5c,d shows the OPLS model that describes metabolite changes observed due to location in the plant irrespective of tissue or genotype.

**Figure 6:**
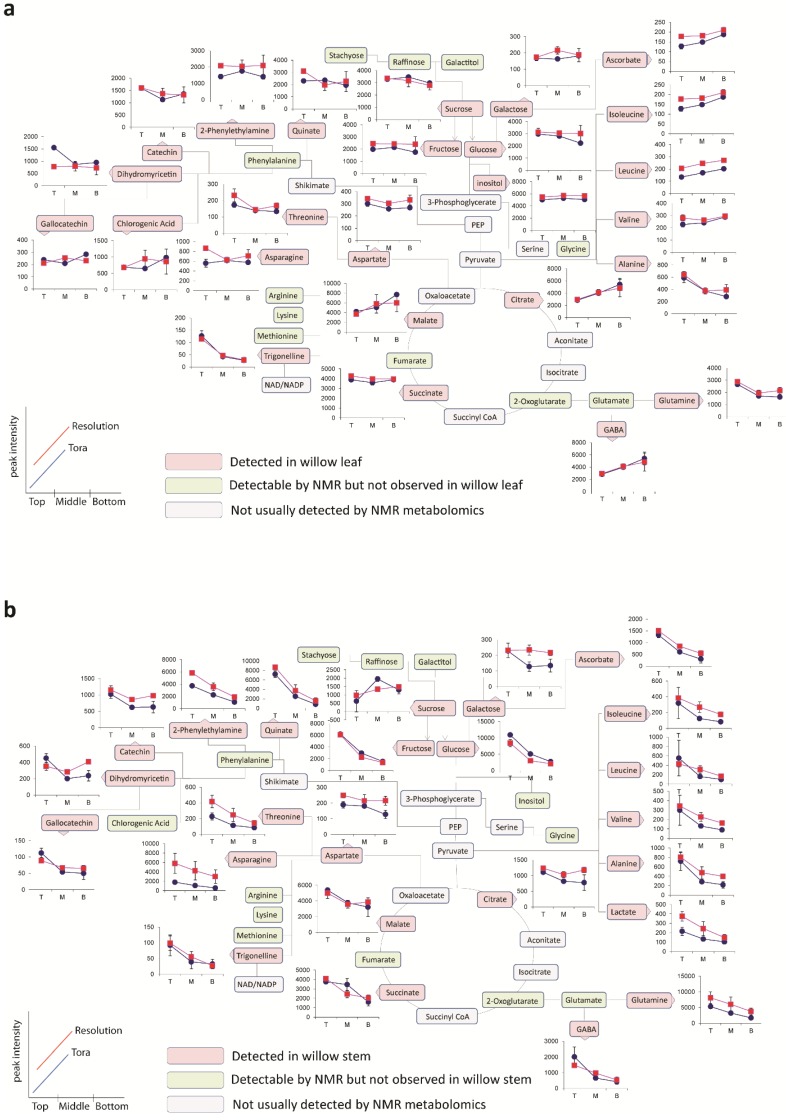
Metabolite trajectories for (**a**) leaf and (**b**) stem samples. Data generated from 1D ^1^H-NMR data using binned regions for characteristic peaks for each metabolite. Plot intensities represent the intensity value of the binned region.

As can be seen from the S-line plot in Figure 5d, a large number of signals are negative indicating that the abundance of the majority of extractable polar metabolites is typically higher in young leaves and stems taken from the top of the plant. Metabolites which oppose this, and that have higher concentrations in older tissue from the base of the plant, include sucrose, citrate and malate. Finally, the model constructed to describe generic differences between Tora and Resolution genotypes in shown in Figure 5e,f. Resolution typically contains higher levels of glutamine, asparagine, 2-phenylethylamine, glutamate and quinic acid. In contrast, Tora samples are generally higher in the major carbohydrates sucrose and glucose. In addition, dihydromyricetin, the major flavonoid in these samples is elevated in the Tora genotype. The PCA and O-PLS models demonstrate that utilising the new extraction protocol, samples from different willow genotypes, where tissue has been obtained from different locations of the plant, can be separated on the basis of their tissue type, harvest point and genotype. O-PLS S-plots detail the major metabolites responsible for these separations. However, it was difficult to ascertain which quantitative metabolite profiles across the sampling position of the plant were able to discriminate the genotypes and which, if any, showed contrasting profiles in the leaf *versus* stem tissue. Figure 6 shows the metabolite trajectories across the height of the plant allowing differences in the profiles to be more easily discerned. In leaves, metabolite profiles (Figure 6a) which discriminate Tora from Resolution include those of leucine, aspartate and 2-phenylethylamine. These metabolites show a similar trajectory but are typically more abundant in one genotype compared with the other. For other metabolites a difference between genotypes can be seen when tissue is harvested from a particular position of the plant. Clear differences in dihydromyricetin levels are observed when leaves are harvested from the top of the plant, but older leaves from the lower part of the plant are unable to discriminate the genotypes. Similar observations are seen for aspartate and glucose. In general, the major soluble carbohydrate concentrations decrease as leaves are sampled from the top to the bottom of the plants while organic acid concentrations (malic and citric) are higher in the lower older leaves. Similarly, the amino acids GABA, glutamine, valine, isoleucine and leucine show higher concentrations in these older leaves from the base of the plant. Contrastingly, alanine, glutamine and threonine levels reach their highest concentration in samples from the top of the plant. Figure 6b shows the same type of metabolite profiles obtained from stem tissue. As suggested by the O-PLS plots, the extracted levels of many metabolites decrease in stem tissue obtained from the lower part of the plant. In many cases, although the profile follows the same trajectory the intensity of the profile is greater in material sampled from Resolution and examples here include asparagine, 2-phenylethylamine, threonine, isoleucine, lactate and glutamine. From this dataset the only metabolite that consistently increased when sampling the lower part of the stem was sucrose. This is in contrast to the profile observed in the leaves where sucrose was typically at its highest level when material was sampled from the top of the plant. Similarly the profiles of many amino acids and organic acids show contrasting profiles in the leaf and stem samples.

The data described in Figure 6 was obtained via scaling the 1D ^1^H-NMR dataset to a known concentration of internal standard (d_4_-TSP) which was present in the extraction solvent. Since 1D ^1^H-NMR is a quantitative technique, irrespective of metabolite chemistry, scaling to the internal standard gives information regarding the absolute concentration of metabolite extracted from 15 mg of dried plant sample. However, from the data in Table 2 we know that the total amount of extractable metabolites is not consistent across all samples in the experiment. Whilst the mass of the soluble metabolome is fairly consistent in leaves and from stem samples obtained from the top of the plant, the amount of extractives obtained from older basal stem sections is considerably lower.

**Figure 7:**
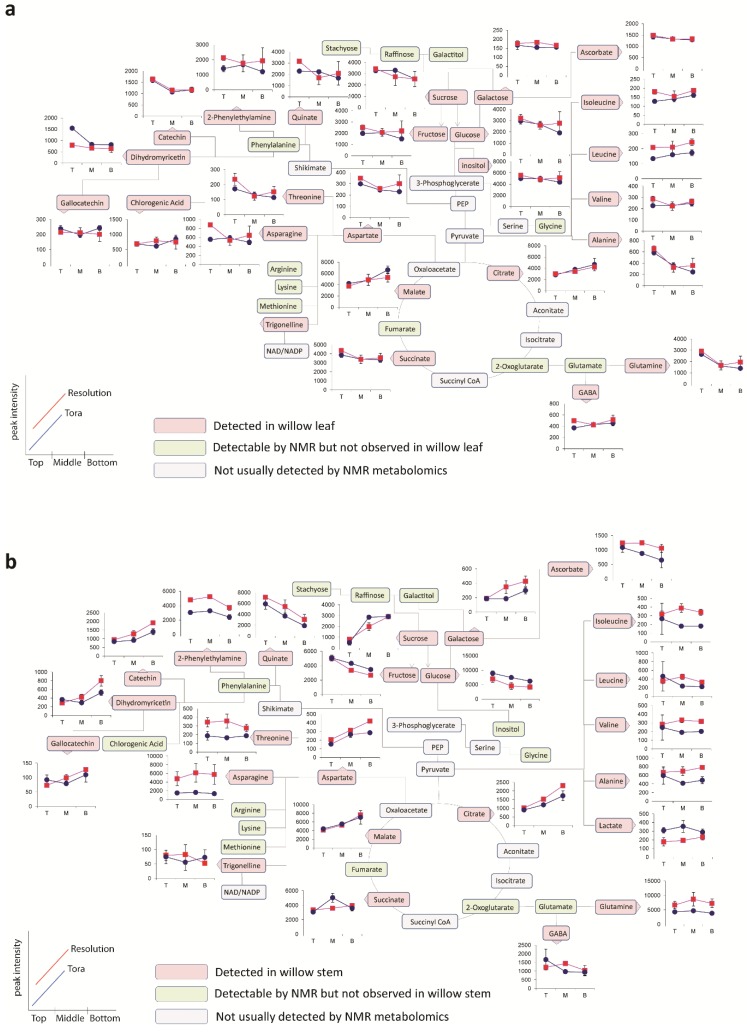
Trajectories for (**a**) leaf and (**b**) stem samples representing changes within the extractable metabolite pool. Data generated from 1D ^1^H-NMR data using binned regions of characteristic peaks for each metabolite which were normalized back to a comparable 3 mg extractable pool weight. Plot intensities represent the intensity value of the normalized binned region.

Thus, while Figure 6 gives an overall picture of levels of each metabolite in each sample, it cannot describe relative changes within the soluble metabolite pool since some of these changes may be masked via a larger change in extractive yield. The new protocol described in this paper, incorporating a measurement of the extractives after dry down, allows the metabolomic 1D ^1^H-NMR data to be normalised to a constant sample weight. This reveals the spatial variation in the dataset allowing metabolite changes within the soluble metabolite pool to be discerned. Figure 7 shows the effect of normalising the data back to a constant 3 mg weight of extractable material. The effect of the normalisation does not alter the direction of the leaf profiles (Figure 7a). This is to be expected since leaves harvested from different parts of the plant typically yielded the same amount of extractable metabolites. However, Figure 7b shows the effect of the normalisation of the stem data. Unlike the data displayed in Figure 6b, which described the diminishing concentrations of the majority of soluble metabolites down the stem, this plot now shows a range of contrasting profiles and represents the real soluble metabolite changes happening within the part of the tissue, irrespective of a changing, and presumably increasing, non-extractable portion of the tissue sample. There is an approximately three-fold difference in the amount of extractives obtained between stems sampled from the top and bottom parts of the plant. Thus, the profile of any metabolite change which is within a three-fold difference may reverse its trajectory when normalised. Those which showed greater than three-fold changes will continue to show the same trajectory although the magnitude of that difference will be attenuated. For the abundant soluble carbohydrates (glucose, fructose and sucrose) the profiles show a similar trajectory to that previously described. However, there has been a large effect on the malate and citrate profiles which now show that both these metabolites actually increase in concentration *within* the soluble metabolite pool as sampling proceeds from the top to the bottom of the plant. Similarly, we see that secondary products such as catechin, gallocatechin and dihydromyricetin increase in stem tissues obtained from the lower portion of the plant. In terms of differences between genotypes, the normalisation of the dataset to a constant weight of extractable metabolites shows that one of the largest differences in profile intensity is now observed for the asparagine content in stems which is very clearly higher in the material sampled from Resolution.

Examination of the direct infusion ESI-MS data from the top, middle and bottom sections of the two genotypes using PCA of the concatenated positive and negative ion spectra revealed that the data shape is in line with that seen for the 1D ^1^H-NMR profiles (Figure S2). Leaf and stem samples could be easily separated in the direction of PC1 (45%) while PC2 (25%) separated the stem data based on sampling location (Figure S2a). When coloured by genotype, PC4 (5%) separated the stem data based on genotype (Figure S2b) and PC5 (1%) discerned differences due to genotype in the leaf samples (Figure S2c).

When PCA models were constructed using stem or leaf data alone, the data further mirrored the clustering observed in PCA of the 1D ^1^H-NMR data (Figure 4). In leaves (Figure S2d), PC1 (81%) described the separation due to sampling point while PC2 (9%) separated the two genotypes. Samples taken from the top of the two different genotypes were easily differentiated. For the stem data only, (Figure S2e), the ESI-MS data again mirrored the 1D ^1^H-NMR data (Figure 4e) with harvest location described by PC1 (58%) and genotype described in the direction of PC2 (32%). Interestingly, it was more difficult to separate samples by genotype when material from the top of the plants was analysed by ESI-MS compared to samples taken from older, lower parts of the plant.

This mirrored the observations from the PCA models constructed from stem 1D ^1^H-NMR data (Figure 4e). Contrastingly, in the leaf only ESI-MS PCA model (Figure S2d), the separation between middle and bottom harvest points was less discernible, when compared to the corresponding 1D ^1^H-NMR PCA model (Figure 4d). However, on the whole the shape of the ESI-MS data matched that of the 1D ^1^H-NMR data, demonstrating that correlation of 1D ^1^H-NMR signal *versus* ESI-MS signal is a valid strategy for metabolite annotation.

### 2.3. Construction and Application of a Bespoke Willow 1D ^1^H-NMR Spectral Library for Automated Quantitation of Metabolites

Provision of a list of metabolites in a sample with their concentrations is the output of choice for multidisciplinary projects where the data is to be mined against other trait or omics datasets or passed onwards for further statistical processing. The nature of 1D ^1^H NMR data and the complexity of typical plant extract spectra with many overlapping peaks from multiple metabolites make manual quantitation difficult and time consuming. Chenomx NMR suite is a set of tools for identifying and quantifying metabolites from 1D ^1^H-NMR spectra of mixtures [48], allowing for quantitation of metabolites even when some signals are overlapped with those from another metabolite. Matching and quantitation can be carried out in automation based on comparison to a library of pH sensitive signatures of authentic metabolites run at differing instrument field strengths. However, as it was developed for clinical metabolomics, the Chenomx library does not contain many common plant metabolites, especially the species specific secondary metabolites. Furthermore, there is no capacity to compare spectra which have been collected in D_2_O:CD_3_OD mixtures. While this was a problem with some earlier versions of the software, Version 7.6 allows users to build user-defined signatures based on their own extraction protocol and 1D ^1^H-NMR data collection parameters. We have therefore constructed a library of signatures from all the abundant primary metabolites detected in Tora and Resolution willow leaves and stems and have supplemented this with signatures from key secondary metabolites such as flavonoids and phenolics and their glycosides, such as salicin and salicortin and triandrin, which are well documented in the *Salix* literature. To date, this bespoke library contains 90 signatures, 52 of which overlap perfectly with those obtained when using the newly developed protocols described above. As an example, matching and quantitation (in μmoles/g dry weight and mg/g dry weight) of the Tora and Resolution leaf and stem data was evaluated and is detailed in Tables S1 and S2. As can be seen by comparison with the data in Figure 6, the use of the Chenomx profiling software has increased the number of metabolites that we were able to quantify. As a means of comparison to the relative data obtained from binning, quantified data in mg/g d.w. have been plotted across tissue types in Figures S3–S6. The profiles of these concentrations agrees well with the majority of metabolites following the same trajectory as that obtained from plotting characteristic regions from the 1D ^1^H-NMR directly. Based on this quantified metabolite data, metabolites showing significant (*p* < 0.05) differences between the Tora and Resolution genotypes could be identified in both stem and leaf tissues sampled at each part of the plant (Table 4).

**Table 4:** Metabolites showing a statistically significant difference between genotypes at varying parts of the willow plant in both leaf and stem tissue. Data is derived from a one-way ANOVA analysis of Chenomx-quantified metabolite concentrations derived from 1D ^1^H-NMR data.

Differentiating Metabolite	*p*-value	Differentiating Metabolite	*p*-value	Differentiating Metabolite	*p*-value
*Top of the plant*		*Middle of the plant*		*Bottom of the plant*	
**Leaves**					
Methionine	<0.00001	Salicin	<0.00001	Methionine	<0.00001
Triandrin	<0.00001	Uridine	0.00052	Lysine	0.00004
Asparagine	0.00005	Asparagine	0.00061	Tyrosine	0.00006
Raffinose	0.00013	3-Hydroxymandelate	0.00094	Triandrin	0.00009
Uridine	0.0003	Leucine	0.00276	Lactic acid	0.00027
Citrate	0.00044	Glutamate	0.0028	Uridine	0.0004
Arginine	0.00065	Maltose	0.00307	Stachyose	0.00053
Dihydromyricetin	0.00138	2-Phenylethylamine	0.0033	Glutamate	0.00071
Stachyose	0.00141	Gamma Aminobutyric acid	0.00404	Sucrose	0.00102
Succinate	0.00174	Glycine	0.00422	Leucine	0.00398
Tyrosine	0.00243	Succinate	0.00727	Asparagine	0.00531
Galactose	0.00254	Lactic acid	0.01097	Arginine	0.00963
Leucine	0.00405	Sucrose	0.01543	Succinate	0.01145
3-Hydroxymandelate	0.00506	Dihydromyricetin	0.02112	Maltose	0.01511
2-Phenylethylamine	0.00722	2-Hydroxyisobutyrate	0.02151	2-Phenylethylamine	0.01618
Lysine	0.01531	Chlorogenic Acid	0.02225	3-Hydroxy-3-methylglutarate	0.02667
Maltose	0.02151	Tyrosine	0.02266	Gamma Aminobutyric acid	0.02981
Quinate	0.02193	Glutamine	0.02382	Acetate	0.03418
Salicin	0.0232	Stachyose	0.03126	Aspartate	0.03775
Glycine	0.036	Fumarate	0.03775		
Chlorogenic acid	0.03749				
Malate	0.04741				
					
**Stems**					
Lactate	<0.00001	Arginine	0.000137	Uridine	0.00016
Stachyose	0.00002	Raffinose	0.000181	Stachyose	0.000274
Succinate	0.00038	Sucrose	0.001103	Succinate	0.001132
Glycine	0.00044	Uridine	0.001187	Arginine	0.00196
Leucine	0.00057	Stachyose	0.002002	Glycine	0.003298
Tryptophan	0.00123	Lysine	0.00206	Methionine	0.003722
Raffinose	0.00133	Methionine	0.002868	Acetate	0.004415
Salicin	0.00143	Tyrosine	0.005116	Trigonelline	0.01361
Uridine	0.00208	Trigonelline	0.005893	Triandrin	0.01569
Dihydromyricetin	0.00371	Maltose	0.006116	Raffinose	0.01611
		Glutamate	0.007043	Leucine	0.01977
		Dihydromyricetin	0.008189	Salicin	0.01997
		2-Phenylethylamine	0.008416	Lysine	0.02147
		Glycine	0.008923	Valine	0.02848
		Choline	0.01115	Lactic acid	0.02986
		Gamma Aminobutyric acid	0.0154	Betaine	0.0316
		Citric acid	0.02168	Maltose	0.0436
		Leucine	0.02354		
					

There is surprisingly little published comparative quantitative data on *S. viminalis* primary metabolites and thus it is difficult to compare the levels of individual metabolites or compound classes found in our study. Some other diverse Salix genotypes have been studied although often these studies have been sampled at different points in the developmental cycle, on other tissue types and are often subject to stresses or heavy metal treatments. Such examples include the assessment of amino acids in phloem and xylem of Salix species [49,50]. In the case of soluble sugars, glucose, sucrose and fructose have been described as the major soluble carbohydrates present in hydroponically grown, juvenile *S. viminalis* leaves [51] where levels reached 35 mg/g d.w. for glucose, 12.5 mg/g d.w. for fructose and 44 mg/g d.w. for sucrose. Our data from field grown tissue mirrors the profile in that glucose and sucrose levels were similar to each other in leaves harvested from the top of the plant and that fructose levels although still abundant were somewhat lower in concentration. The overall concentration of leaf soluble sugars appears lower in older field grown material compared to that reported for young plants. This is in agreement with data presented on *Populus deltoides* × *nigra* where similar levels of carbohydrates were reported to our own study [52].

In terms of organic acids, malate, citrate, ascorbate and quinate levels dominated the organic acids fraction of leaves in our study while major components in stems were ascorbate, malate, quinate and 2-oxoglutarate, the latter being highest from stem material harvested from the top of the plant. Malate and citrate levels (on a fresh weight basis) are reported in leaves of *S. alba* at 1.6 and 0.6 mg/g F.W. respectively [53]. Thus, our observations of 3–10 mg/g d.w. of citrate in leaves are broadly comparable. Similarly, results of 6–22 mg/g d.w. of malate in *S. viminalis* are comparable with levels observed on a fresh matter basis in *S. alba* leaves. Willow and poplar are well known for the diversity of phenolic glycosides present in stem tissues [54], although it is also recognized that levels of such metabolites vary over the growth season [55]. *S. viminalis* tissue is typically low in the salicinoids, during periods of active growth, compared to other varieties of willow such as *S. purpurea* [56]. Thus, as expected, we observed only small amounts of salicin (typically <1 mg/g d.w.) in this experiment. Additionally, the 1,4-substituted analogue triandrin was detected in all leaf and stem samples, consistent with previous findings [56] that it is a common component in *S. viminalis*. The aromatic regions of our spectra also contained a mixture of flavanols, with major components such as dihydromyricetin, catechin and gallocatechin. Levels of these compounds in our study ranged from 0.23–7 mg/g d.w. Such high levels of these compounds have previously been reported in stem tissues of e.g., *S. caprea* [57].

**Figure 8:**
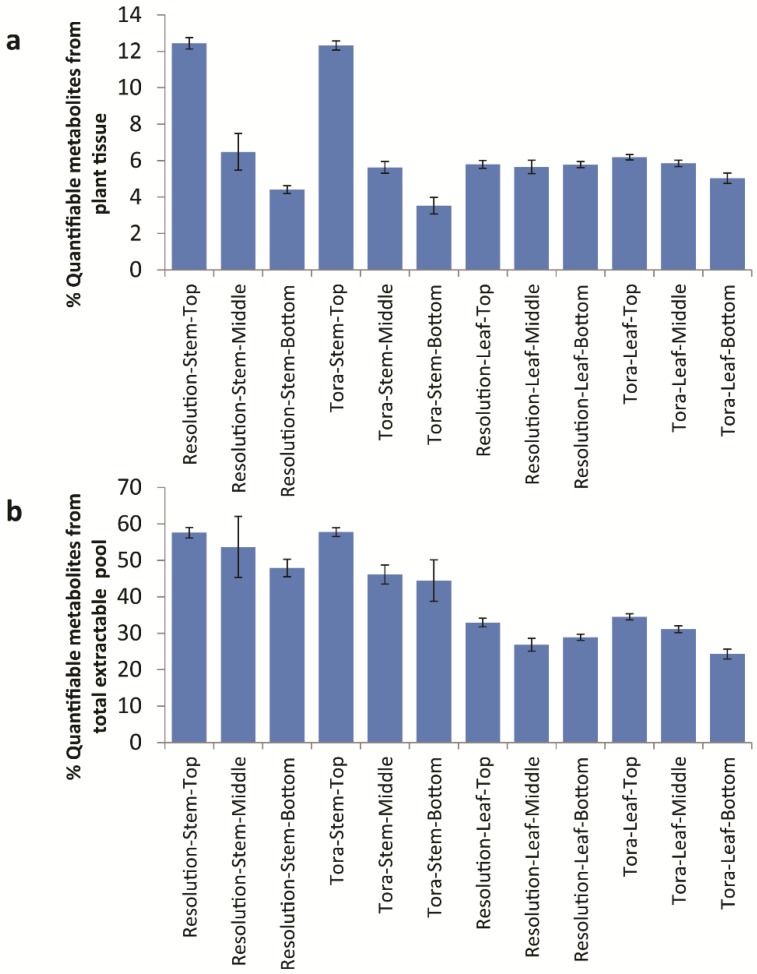
Calculated quantifiable metabolites (%) as a proportion of (**a**) total plant tissue and (**b**) total soluble metabolite pool. Data obtained from Chenomx quantification of metabolites as measured by 1D ^1^H-NMR.

**Figure 9:**
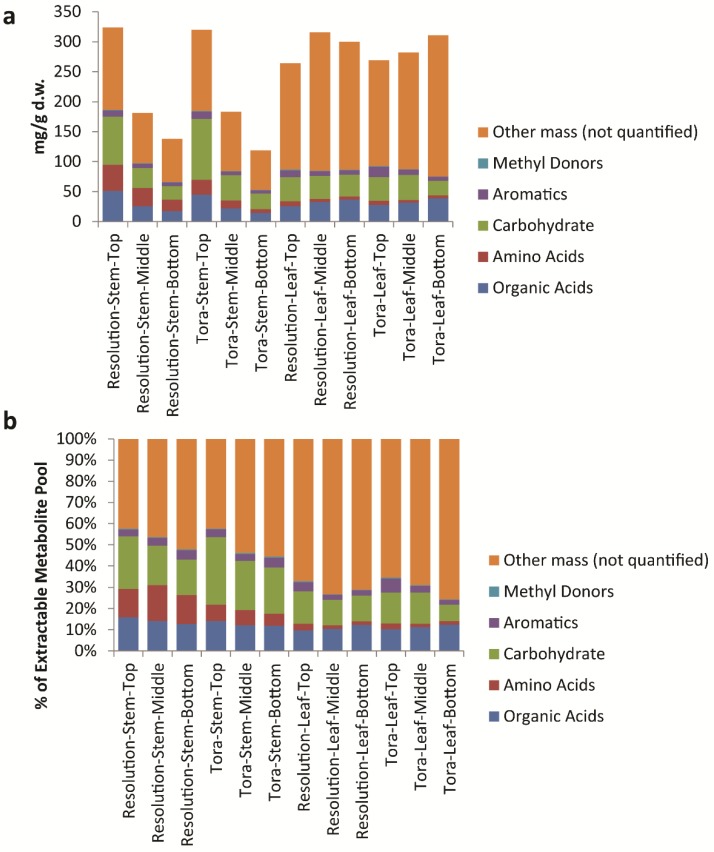
Calculated levels of compound classes via Chenomx quantification of metabolites as measured by 1D ^1^H-NMR. (**a**) Class levels expressed as mg/g dry weight; (**b**) expressed as a percentage of the total soluble metabolite pool.

Conversion of quantified data to units of mg/g d.w. allowed a total concentration of quantified metabolites to be elucidated (Table S2). Of note here is the fact that, in leaf, the concentration of total quantified metabolites ranged from 75 mg/g d.w. to 93 mg/g d.w. and did not vary significantly by genotype or tissue position. This is in parallel with the data outlined in Table 2 relating to the variation in % extractable metabolites from leaf. However, 90 mg/g d.w. of quantified metabolites in leaf samples represents approximately 30% of the known extractable mass. Thus, in leaves, ~70% of polar extractives relate to unknowns that either have not yet been quantified or to substances that do not give signals in the 1D ^1^H-NMR spectrum (Figure 8). Examples here would be inorganics such as phosphate, metal salts or oxalate (which is known to be high in willow leaves, [29]) or multiple low abundance metabolites that are below the level of detection in NMR. From Chenomx assignments, it is the latter which is most likely. When compounds are examined by their chemical classes (Figure 9), it is clear to see that the only class that changes in the absolute amount per gram of leaf tissue is the organic acids which are at their highest level in older leaves at the bottom of the plant.

When metabolite concentrations were normalised to the metabolite pool, we can also see that total levels of amino acids, carbohydrates and aromatics are highest in young leaves from the top of the plant. In contrast, mass that is 1D ^1^H-NMR invisible such as inorganic salts is lowest in young leaves. In stem tissues the absolute amount of metabolites that can be quantified per gram of plant tissue decreases (Figure 8). However, within the pool the % of these quantifiable metabolites is relatively static. In terms of 1D ^1^H-NMR invisible metabolites, these are lowest in material from the top of the plant and increase in older stem tissue, although even here the mass of such metabolites is lower than seen in leaf material (Figure 9). In terms of stem organic acids, these show a similar behaviour in both genotypes with highest levels at the top of the plant. In contrast to leaves, organic acid concentrations are lowest from stem material collected from the bottom of the plant. Levels of total soluble carbohydrates and amino acids discriminate genotypes with Tora containing higher stem carbohydrate and Resolution higher stem amino acids. Total aromatic metabolites are similar in both genotypes with highest levels of these compounds isolated from younger tissue. The development of the Chenomx metabolite library in concert with the methods described for sample handling and data collection therefore enable a detailed list of metabolites to be generated in high throughput for comparison of metabolite pools and compound classes between samples and will enable future large scale metabolomics experiments, such as mQTL studies, in willow.

### 2.4. Simplification of the Method for High Throughput 1D ^1^H-NMR-MS Screening

**Figure 10:**
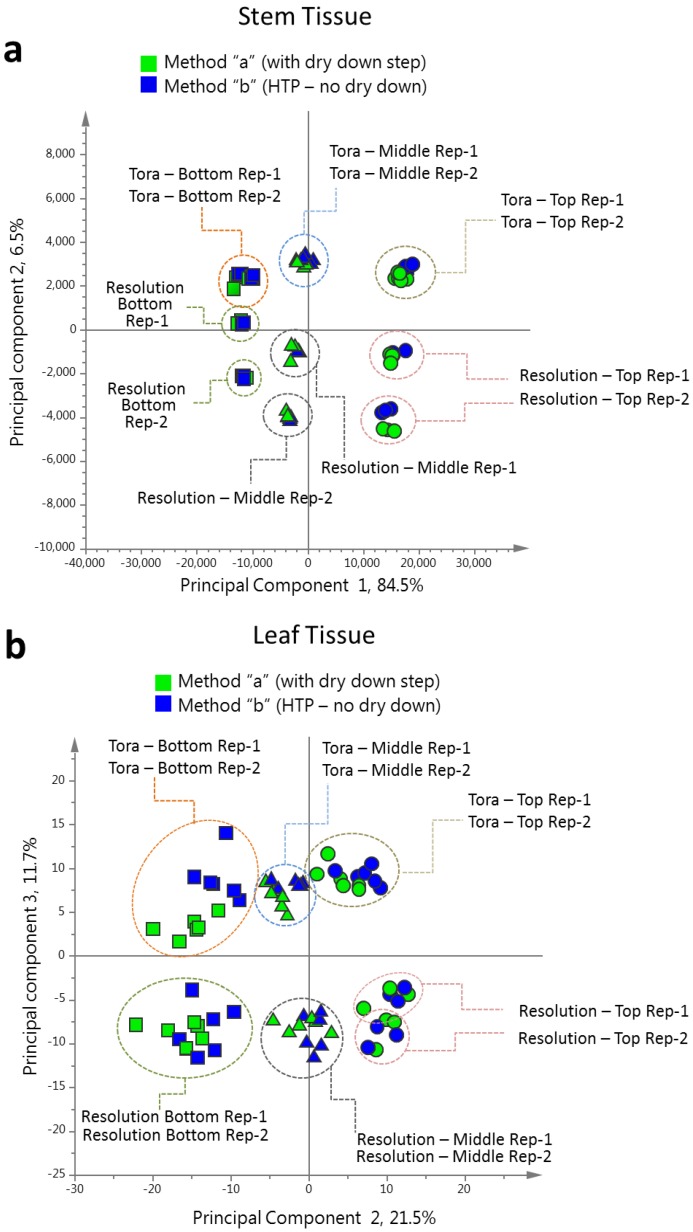
Comparison of binned 1D ^1^H-NMR data from extracts prepared by method “a” and method “b” (Figure S7). (**a**) PCA scores plot of willow stem 1D ^1^H-NMR data coloured by method used to prepare NMR extracts; green: method “a”; blue: method “b”. (**b**) PCA scores plot of willow leaf 1D ^1^H-NMR data coloured by method used to prepare NMR extracts; green: method “a”; blue: method “b”.

Above, after much optimisation we developed a robust protocol for 1D ^1^H-NMR-MS screening of the willow metabolome. The protocol (Figure S7a) was developed and deployed above with a dry-down step, for recording of extractable weight, allowing normalisation and study of the dynamics of the metabolite pool. However, for the large-scale screening of comparable tissues from genetic populations for mQTLs, where wet-lab processing steps are ideally kept to a minimum, the method was modified according to Figure S7b and the final entry of Table 1. Tissue was extracted directly into deuterated NMR solvent and the dry down/reconstitution step was removed. After removal of aliquots for ESI-MS, NMR samples were then modified with pH 7.4 phosphate buffer and EDTA, prior to spectral data collection. Analysis of the resultant 1D ^1^H-NMR spectra showed that samples prepared without the dry down step contained higher levels of ascorbate and acetate. These were the only evident changes between the two methods. Comparison of the data, obtained by the two methods, by PCA (Figure 10) showed that corresponding samples prepared by each method still clustered together and that the separation by harvest position or genotype was larger than any difference between the two modes of extract preparation.

## 3. Experimental Section

### 3.1. Plant Material

Tissue from the two biomass varieties, Tora and Resolution, was harvested from the National Willow Collection at Rothamsted in June 2012 (Figure S4). Both genotypes are *Salix viminalis* × *S. schwerinii* hybrids and are female and diploid. They are distantly related in that a sibling of Tora (Bjorn) is the male parent of both parents of Resolution. The original planting of Tora was in 2002, whilst that of Resolution was 2004. The plots had previously been coppiced in February 2012 and thus the material represented *circa* 4 months fresh regrowth from stools. The freshly coppiced plots had been treated with herbicide (amitrole, 20 L/ha) and nitrogen fertiliser in February 2012. Immediately after harvest, leaves and stems from each genotype were each divided into three samples representing bottom (1–30 cm), middle (31–60 cm) and top (61 cm and above) parts of each plant. Two similar sized plants were harvested and dissected thus producing two biological replicates of each genotype/tissue type. Samples were frozen in liquid nitrogen, then freeze-dried and milled to a powder in a cryo-mill. They were stored at −80 °C prior to analysis.

### 3.2. Preparation of NMR-MS Samples for Willow, Incorporating a Dry-Down Step for Determination of Mass of Extracted Metabolites

To triplicate aliquots (15.0 mg) of each freeze-dried, milled plant sample in 2 mL round bottom Eppendorf tubes, was added H_2_O-CH_3_OH (4:1) extraction solvent (1.0 mL). After mixing, the tubes were heated to 50 °C for 10 min, cooled and centrifuged. From each tube, supernatant (850 μL) was transferred to a clean Eppendorf tube and then heated to 90 °C for 2 min. The samples were then cooled to 4 °C for 30 min and then centrifuged. For ESI-MS, 50 μL of the supernatant was removed to a glass HPLC vial and diluted with 950 μL of H_2_O-CH_3_OH (4:1). For extract mass determination and subsequent 1D ^1^H-NMR analysis, 700 μL of the supernatant were transferred to a clean pre-weighed Eppendorf tube and then evaporated in a vacuum concentrator overnight at 30 °C. After further drying (30 min) in a vacuum oven (room temperature), the weight was recorded and 700 μL of NMR solvent [D_2_O-CD_3_OD, 4:1 v/v, incorporating 0.01% w/v 2,2,3,3-*d*_4_- 3-(trimethylsilyl)propionic acid (TSP)] was added. After dissolution at room temperature, 20 μL deuterated 2.6 M phosphate buffer, pH 7.4 [containing 4.19 g K_2_HPO_4_ and 0.808 g KH_2_PO_4_ in 10 mL D_2_O] was added along with 10 μL of EDTA solution [32 mM, containing 12 mg ETDA-Na_2_.2H_2_O in 1 mL D_2_O]. After mixing and standing for 30 min, the samples were centrifuged and 650 μL were removed to clean, dry 5 mm NMR tubes.

### 3.3. Sample Preparation for High-Throughput 1D ^1^H-NMR-MS Screening of Willow, Utilising Direct Extraction into Deuterated NMR Solvent

To triplicate aliquots (15.0 mg) of each freeze-dried, milled plant sample in 2 mL round bottom Eppendorf tubes, was added D_2_O-CD_3_OD (4:1 v/v) incorporating 0.01% w/v TSP (1.0 mL). After mixing, the tubes were heated to 50 °C for 10 min, cooled and centrifuged. From each tube supernatant (850 μL) was transferred to a clean Eppendorf tube and then heated to 90 °C for 2 min. The samples were then cooled to 4 °C for 30 min and then centrifuged. For ESI-MS, 50 μL of the supernatant was removed to a glass HPLC vial and diluted with 950 μL of H_2_O-CH_3_OH (4:1). For NMR, 700 μL of the supernatant was removed to a clean Eppendorf tube and mixed with 20 μL deuterated 2.6 M phosphate buffer, pH 7.4 and 10 μL of 32 mM EDTA solution in D_2_O, as above. 650 μL of this buffered sample was transferred to a 5 mm NMR tube.

### 3.4. 1D ^1^H-NMR and Direct Infusion ESI-MS Data Collection and Data Analysis

These were respectively carried out on an Avance 600 MHz NMR Spectrometer (Bruker Biospin, Coventry, UK) and an Esquire 3000 mass spectrometer (Bruker Daltonics, Coventry, UK) using parameters and settings as previously described [30]. Briefly, 1D ^1^H-NMR spectra were acquired at 300 K using a 5 mm SEI probe. A water suppression pulse sequence (noesygppr1d) was utilised employing a 90° excitation pulse angle and a pre-saturation pulse during the relaxation delay of 5 s. Data were acquired using 128 scans of 65,536 data points across a sweep width of 12 ppm. 1D ^1^H-NMR FIDs were zero filled to double their original size, and Fourier transformed with an exponential window function (0.5 Hz). Spectra were manually phased and automatically baseline corrected in Amix (Analysis of MIXtures, Bruker Biospin) using a 2nd order polynomial. ^1^H chemical shifts were referenced to d_4_-TSP at δ0.00 and spectra were automatically reduced to create an ASCII file containing integrated regions of equal width (0.015 ppm). Spectral intensities were scaled to the d_4_-TSP region (δ0.05 to −0.05). The ASCII file was imported into Excel for the addition of sampling/treatment details. The regions for unsuppressed water (δ4.865–4.775), d_4_-MeOH (δ3.335–3.285) and d_4_-TSP (δ0.05 to −0.05) were removed prior to importing the dataset into SIMCA-P 13.0 (Umetrics, Umea, Sweden) for multivariate analysis. Multivariate analysis (PCA and OPLS) was carried out using unit variance scaling. For construction of trajectory plots of individual metabolites, data from characteristic regions for known metabolites was combined to give a single intensity response for each metabolite. Technical replicates were averaged and errors displayed on the basis of 2 biological replicates. Annotation of peaks to individual metabolites was achieved via comparison to a library of authentic standards prepared in identical conditions to the test samples and run under identical 1D ^1^H-NMR conditions.

### 3.5. Automated Batch Quantification of Target Metabolites

Batch quantification of metabolites in 1D ^1^H-NMR spectra was achieved using the Chenomx NMR Suite 7.6 (Chenomx Inc., Edmonton, AB, Canada) [48]. A database of 90 metabolite signatures was built from spectra of authentic pure samples of common plant metabolites and willow-specific secondary products, by collecting spectra at 600 MHz on the same spectrometer and instrument settings in the pH 7.4 and EDTA modified solvent as above. The standard metabolites were quantified against the known concentration of reference compound (TSP) and fitted to record peak centres and coupling constants in the database. Quantitative profiling across the willow batched spectra was carried out using the Profiler module in the software, which superimposes a Lorentzian peak shape model for each database entry onto the analyte spectra, and reports a concentration for each matched metabolite in each spectrum. Every metabolite fit was manually inspected. Data for technical replicates were averaged and a mean concentration for each biological sample was tabulated. The output data table was examined by PCA (SIMCA-P, Umetrics, Umea, Sweden), to quality assure the Chenomx determined quantitations by means of the inbuilt biological and technical replication. Significance of metabolite concentration differences was determined using one-way ANOVA and was carried out in Microsoft Excel. A table of characteristic chemical shifts for metabolites identified from Tora and Resolution genotypes has been included as Table S3.

## 4. Conclusions

In summary, we have overcome a variety of technical challenges and developed a robust method for high throughput screening of the willow primary and secondary metabolomes, which gives 1D ^1^H-NMR and ESI-MS data on the same samples with low variation due to technical replication. The method allows direct statistical comparison and correlation of stem (wood) and leaf samples from any part of the willow plant and across the two spectroscopic datasets, and this has been demonstrated *via* a range of statistical methods which are common in many metabolomics studies. The processing regime also allows for measurement of the extractable mass of the soluble metabolome, data that will be necessary for modelling metabolic flow from sources to sinks. A streamlined adaption of the method for high-throughput screening was also refined and demonstrated to be robust.

In addition to the quantification of metabolites via integration of characteristic bins in the processed data, we have automated quantitation of 52 metabolites in the 1D ^1^H-NMR spectra, using Chenomx and show that the results are comparable. Either method enables rapid extraction of quantitative data from high throughput genetic screens, which we are now conducting across the extensive genotype collections held at Rothamsted. We would anticipate that the methods developed here are directly applicable to related species such as poplar, and potentially to many other woody biomass crops. Using samples taken from the two willow genotypes, we have also demonstrated that the 1D ^1^H-NMR and ESI-MS datasets show the same trajectories when modelled by PCA, and thus we expect that meaningful NMR to MS structural information can be gleaned from combined analysis of these two datasets. Furthermore, as NMR is non-destructive, the samples are available for further spectroscopic investigation to follow up on metabolites of interest. We are now applying these methods to diversity and mapping populations, with a view to identifying mQTLs for biomass yield and other agronomic traits, including selection of lines for novel metabolite related properties. Studies in annotation of the ESI-MS data are also underway, including a very high resolution uHPLC-ESI-MS-MS study to further enhance the value of the screen. Details of this study will be published elsewhere.

## Supplementary Files

Supplementary File 1
