# Supplementary material for: An Efficient High Throughput Metabotyping Platform for Screening of Biomass Willows
Source: Metabolites. 2014 Oct 28;4(4):946–76. doi: 10.3390/metabo4040946 (PMC4279154; doi:10.3390/metabo4040946)

Figure S1. PCA scores plots of 1 D ^{1}H - NMR data, indicating clustering of Tora and Resolution leaf and stem samples. Indication within the coloured clusters is highlighted to show clustering of the three technical replicates. **(a)** PC1 vs PC2 of leaf data only, coloured by genotype and harvest position; **(b)** PC1 vs PC2 of stem data only, coloured by genotype and harvest position. Harvest position: B:bottom; M:middle; T:top.

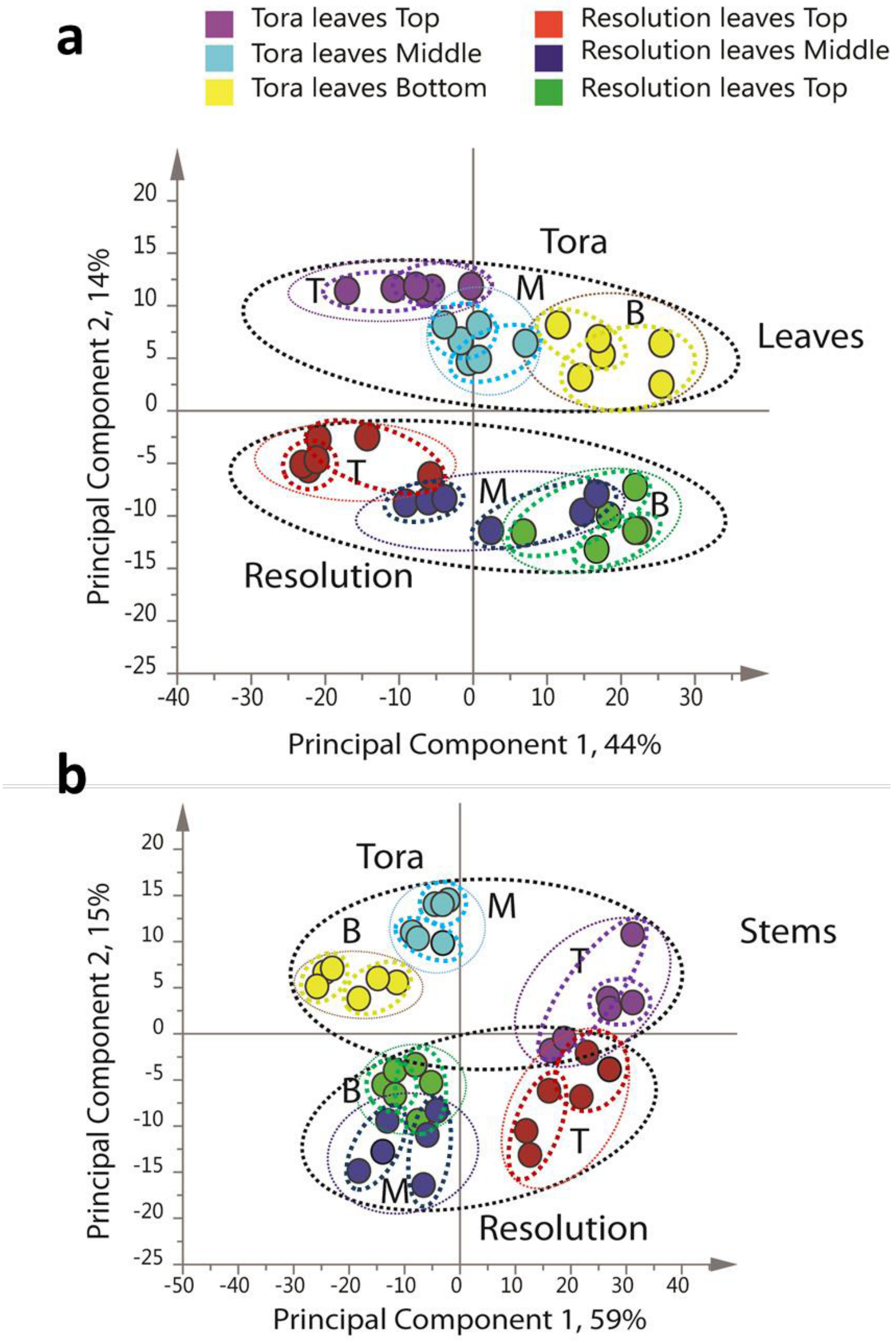

Supplement: Supplementary File 1 [file metabolites-04-00946-s001.zip › metabolites-64902-sup-update/Figure S1.pdf]
