# Supplementary material for: An Efficient High Throughput Metabotyping Platform for Screening of Biomass Willows
Source: Metabolites. 2014 Oct 28;4(4):946–76. doi: 10.3390/metabo4040946 (PMC4279154; doi:10.3390/metabo4040946)

Figure S2. PCA scores plots of concatenated positive and negative mode ESI-MS data, indicating clustering of Tora and Resolution leaf and stem samples. a: PC1 vs PC2 of leaf and stem data, coloured by harvest position; b: PC1 vs PC4 of leaf and stem data, coloured by genotype; c: PC1 vs PC5 of leaf and stem data, coloured by genotype; d: PC1 vs PC2 of leaf data only, coloured by genotype and harvest position; e: PC1 vs PC2 of stem data only, coloured by genotype and harvest position

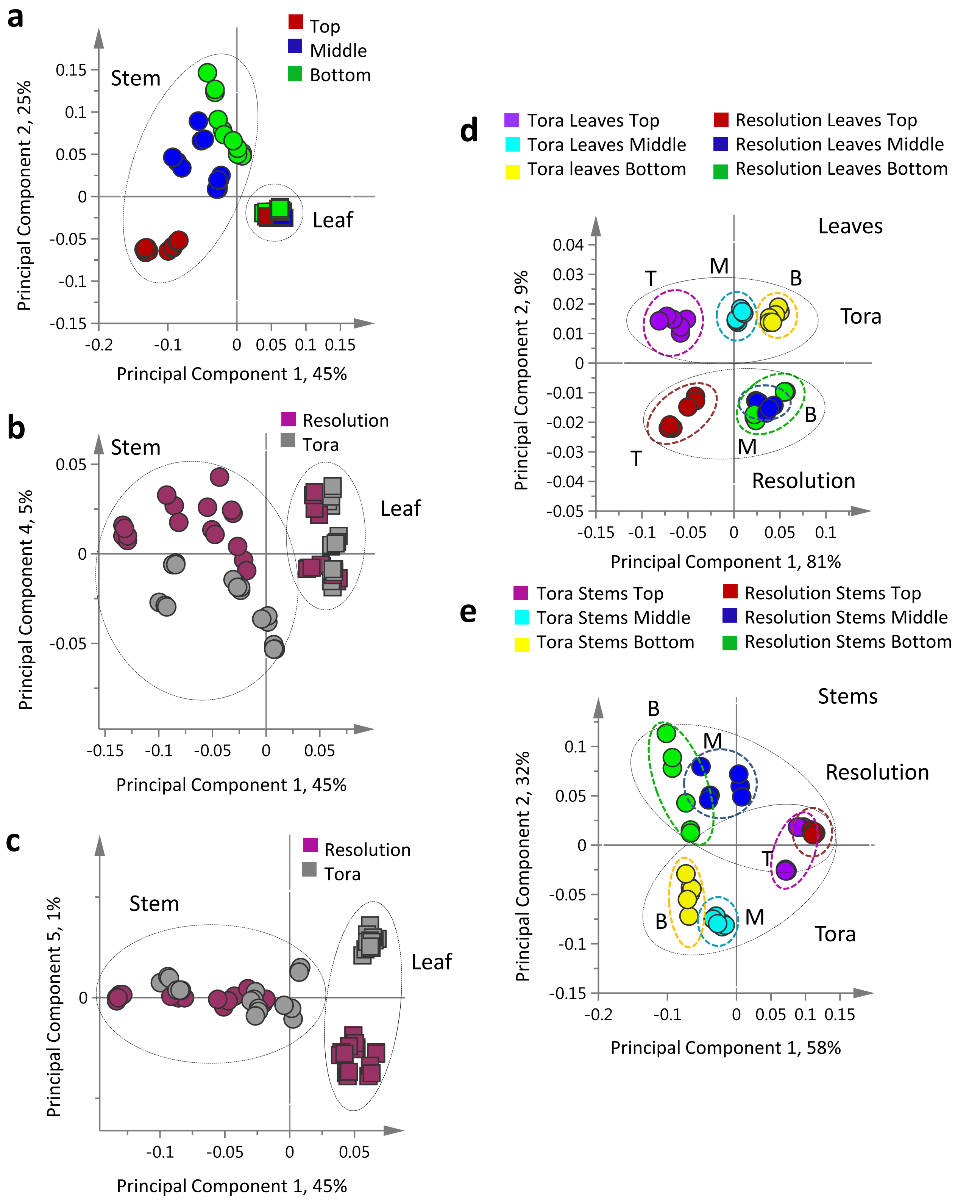

Supplement: Supplementary File 1 [file metabolites-04-00946-s001.zip › metabolites-64902-sup-update/Figure S2.pdf]
