# Supplementary material for: An Efficient High Throughput Metabotyping Platform for Screening of Biomass Willows
Source: Metabolites. 2014 Oct 28;4(4):946–76. doi: 10.3390/metabo4040946 (PMC4279154; doi:10.3390/metabo4040946)

Figure S5: Quantified data (via Chenomx) from 1D ^{1}H NMR of willow stem tissue normalised to the extracted metabolite pool. Concentrations given in micrograms per mg of the extactable pool. Data points correspond to mean ± standard deviation from two biological replicates. Red = Resolution; Blue = Tora. T = material harvested from the top of the plant. M = material harvested from the middle of the plant. B = material harvested from the bottom of the plant.

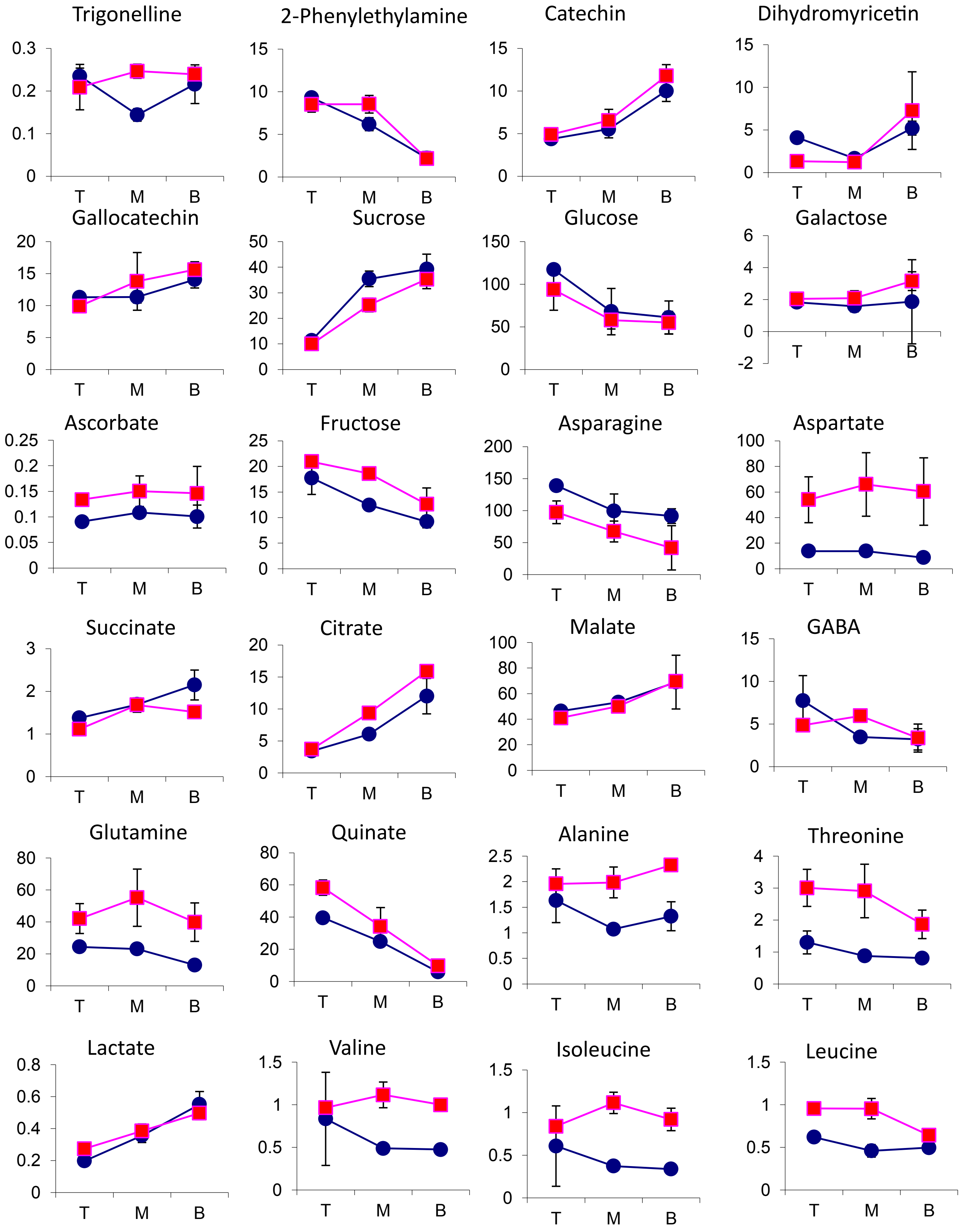

Supplement: Supplementary File 1 [file metabolites-04-00946-s001.zip › metabolites-64902-sup-update/Figure S5.pdf]
