# Supplementary material for: An Efficient High Throughput Metabotyping Platform for Screening of Biomass Willows
Source: Metabolites. 2014 Oct 28;4(4):946–76. doi: 10.3390/metabo4040946 (PMC4279154; doi:10.3390/metabo4040946)
Supplement: Supplementary File 1 [file metabolites-04-00946-s001.zip › metabolites-64902-sup-update/Table S1.docx]

**Table S1.** Concentrations (mg/g d.w.) of 52 metabolites quantified from 1D ^1^H-NMR data of willow leaf and stem tissue *via* Chenomx software against a library of 90 compound signatures run under identical conditions.

| Genotype | **Resolution** | | | **Tora** | | | **Resolution** | | | **Tora** | | |
| --- | --- | --- | --- | --- | --- | --- | --- | --- | --- | --- | --- | --- |
| Tissue | **Leaf** | | | **Leaf** | | | **Stem** | | | **Stem** | | |
| Harvest Position | **Top** | **Middle** | **Bottom** | **Top** | **Middle** | **Bottom** | **Top** | **Middle** | **Bottom** | **Top** | **Middle** | **Bottom** |
| **Carbohydrates** |  |  |  |  |  |  |  |  |  |  |  |  |
| Sucrose | 12.338 ± 0.086 | 12.013 ± 0.079 | 8.881 ± 0.081 | 12.532 ± 0.124 | 12.537 ± 0.048 | 3.992 ± 0.207 | 3.446 ± 0.212 | 4.868 ± 0.065 | 5.131 ± 0.059 | 3.973 ± 0.018 | 6.910 ± 0.069 | 4.939 ± 0.035 |
| Glucose | 12.773 ± 1.484 | 9.732 ± 1.282 | 10.123 ± 3.904 | 10.155 ± 0.197 | 9.609 ± 0.131 | 5.658 ± 0.353 | 32.508 ± 7.608 | 11.109 ± 0.690 | 7.937 ± 1.181 | 41.145 ± 3.823 | 13.035 ± 4.184 | 7.621 ± 1.146 |
| Fructose | 6.807 ± 2.508 | 6.547 ± 1.494 | 7.380 ± 1.406 | 5.492 ± 1.435 | 4.907 ± 0.220 | 3.627 ± 1.211 | 34.371 ± 5.999 | 12.887 ± 1.611 | 5.940 ± 4.403 | 48.627 ± 0.155 | 19.262 ± 3.622 | 11.526 ± 0.352 |
| Maltose | 0.411 ± 0.002 | 0.376 ± 0.000 | 0.438 ± 0.005 | 0.438 ± 0.004 | 0.438 ± 0.004 | 0.389 ± 0.008 | 0.487 ± 0.038 | 0.378 ± 0.000 | 0.340 ± 0.001 | 0.550 ± 0.200 | 0.346 ± 0.002 | 0.229 ± 0.034 |
| Raffinose | 0.505 ± 0.003 | 1.756 ± 1.515 | 0.800 ± 0.065 | 0.723 ± 0.002 | 0.721 ± 0.000 | 0.788 ± 0.003 | 0.723 ± 0.006 | 0.729 ± 0.000 | 0.729 ± 0.000 | 0.234 ± 0.027 | 0.168 ± 0.010 | 0.280 ± 0.079 |
| Stachyose | 0.618 ± 0.008 | 0.331 ± 0.009 | 1.261 ± 0.005 | 0.336 ± 0.014 | 0.815 ± 0.122 | 1.586 ± 0.008 | 0.908 ± 0.000 | 0.913 ± 0.007 | 0.786 ± 0.009 | 0.131 ± 0.001 | 0.301 ± 0.038 | 0.336 ± 0.001 |
| Galactose | 0.294 ± 0.012 | 0.410 ± 0.001 | 0.327 ± 0.100 | 2.211 ± 0.137 | 4.205 ± 2.567 | 0.723 ± 0.513 | 0.707 ± 0.014 | 0.398 ± 0.042 | 0.454 ± 0.044 | 0.639 ± 0.014 | 0.300 ± 0.044 | 0.211 ± 0.299 |
| Fucose | 0.192 ± 0.025 | 0.262 ± 0.130 | 0.228 ± 0.004 | 0.176 ± 0.001 | 0.154 ± 0.010 | 0.134 ± 0.044 | 0.253 ± 0.027 | 0.141 ± 0.048 | 0.134 ± 0.007 | 0.114 ± 0.008 | 0.085 ± 0.030 | 0.135 ± 0.050 |
| **Sugar alcohols** |  |  |  |  |  |  |  |  |  |  |  |  |
| Myo-inositol | 6.165 ± 0.194 | 6.796 ± 0.592 | 6.976 ± 0.854 | 7.210 ± 1.353 | 8.106 ± 0.527 | 7.103 ± 0.435 | 6.899 ± 1.119 | 1.811 ± 0.263 | 1.493 ± 0.698 | 6.431 ± 0.071 | 1.859 ± 0.110 | 0.559 ± 0.791 |
| **Amino acids** |  |  |  |  |  |  |  |  |  |  |  |  |
| Glutamate | 2.569 ± 0.019 | 1.892 ± 0.018 | 1.678 ± 0.002 | 2.296 ± 0.175 | 1.584 ± 0.013 | 1.124 ± 0.021 | 0.946 ± 0.060 | 1.363 ± 0.007 | 1.535 ± 0.025 | 1.349 ± 0.020 | 1.515 ± 0.017 | 1.486 ± 0.012 |
| Glutamine | 2.024 ± 0.068 | 1.084 ± 0.106 | 1.080 ± 0.020 | 2.131 ± 0.206 | 0.610 ± 0.001 | 1.085 ± 0.031 | 14.616 ± 3.728 | 10.914 ± 4.749 | 5.880 ± 2.277 | 8.515 ± 0.606 | 4.534 ± 0.816 | 1.645 ± 0.208 |
| Glycine | 0.536 ± 0.030 | 0.290 ± 0.003 | 0.439 ± 0.017 | 0.427 ± 0.002 | 0.484 ± 0.018 | 0.426 ± 0.011 | 2.931 ± 0.034 | 1.234 ± 0.021 | 0.161 ± 0.007 | 4.123 ± 0.008 | 1.992 ± 0.100 | 1.006 ± 0.068 |
| Alanine | 0.499 ± 0.055 | 0.196 ± 0.056 | 0.188 ± 0.094 | 0.473 ± 0.077 | 0.201 ± 0.039 | 0.074 ± 0.005 | 0.675 ± 0.077 | 0.389 ± 0.104 | 0.339 ± 0.033 | 0.567 ± 0.114 | 0.210 ± 0.019 | 0.171 ± 0.062 |
| Arginine | 0.469 ± 0.001 | 0.375 ± 0.004 | 0.443 ± 0.006 | 0.361 ± 0.004 | 0.377 ± 0.005 | 0.583 ± 0.019 | 0.724 ± 0.000 | 0.831 ± 0.004 | 0.505 ± 0.002 | 0.665 ± 0.006 | 0.595 ± 0.000 | 0.306 ± 0.012 |
| Aspartate | 0.018 ± 0.001 | 0.010 ± 0.001 | 0.019 ± 0.000 | 0.021 ± 0.003 | 0.013 ± 0.000 | 0.022 ± 0.001 | 0.046 ± 0.001 | 0.029 ± 0.002 | 0.021 ± 0.006 | 0.032 ± 0.003 | 0.021 ± 0.000 | 0.013 ± 0.001 |
| Asparagine | 0.721 ± 0.004 | 0.416 ± 0.007 | 0.284 ± 0.002 | 0.285 ± 0.002 | 0.222 ± 0.002 | 0.245 ± 0.002 | 18.825 ± 6.829 | 13.091 ± 6.363 | 8.983 ± 4.634 | 4.805 ± 0.104 | 2.697 ± 0.015 | 1.142 ± 0.516 |
| GABA | 0.117 ± 0.029 | 0.146 ± 0.001 | 0.179 ± 0.008 | 0.093 ± 0.004 | 0.105 ± 0.003 | 0.139 ± 0.006 | 1.676 ± 0.194 | 1.154 ± 0.085 | 0.501 ± 0.285 | 2.667 ± 0.866 | 0.676 ± 0.006 | 0.421 ± 0.223 |
| Isoleucine | 0.124 ± 0.025 | 0.086 ± 0.007 | 0.096 ± 0.005 | 0.081 ± 0.017 | 0.061 ± 0.020 | 0.094 ± 0.084 | 0.290 ± 0.012 | 0.218 ± 0.050 | 0.135 ± 0.031 | 0.207 ± 0.151 | 0.073 ± 0.005 | 0.042 ± 0.001 |
| Lysine | 0.269 ± 0.020 | 0.216 ± 0.028 | 0.184 ± 0.000 | 0.158 ± 0.001 | 0.159 ± 0.002 | 0.303 ± 0.001 | 0.039 ± 0.000 | 0.079 ± 0.001 | 0.141 ± 0.008 | 0.048 ± 0.001 | 0.047 ± 0.000 | 0.175 ± 0.000 |
| Leucine | 0.049 ± 0.003 | 0.045 ± 0.000 | 0.079 ± 0.000 | 0.080 ± 0.000 | 0.057 ± 0.001 | 0.047 ± 0.003 | 0.330 ± 0.000 | 0.184 ± 0.001 | 0.093 ± 0.003 | 0.217 ± 0.004 | 0.090 ± 0.021 | 0.063 ± 0.005 |
| Methionine | 0.051 ± 0.000 | 0.077 ± 0.019 | 0.025 ± 0.000 | 0.074 ± 0.000 | 0.040 ± 0.001 | 0.060 ± 0.001 | 0.414 ± 0.001 | 0.303 ± 0.006 | 0.215 ± 0.010 | 0.400 ± 0.003 | 0.210 ± 0.003 | 0.033 ± 0.012 |
| Threonine | 0.400 ± 0.105 | 0.102 ± 0.029 | 0.122 ± 0.040 | 0.271 ± 0.036 | 0.107 ± 0.003 | 0.054 ± 0.023 | 1.043 ± 0.238 | 0.574 ± 0.230 | 0.275 ± 0.090 | 0.451 ± 0.096 | 0.172 ± 0.021 | 0.103 ± 0.009 |
| Tryptophan | 0.274 ± 0.045 | 0.293 ± 0.046 | 0.280 ± 0.044 | 0.408 ± 0.119 | 0.443 ± 0.051 | 0.443 ± 0.039 | 0.204 ± 0.005 | 0.081 ± 0.007 | 0.045 ± 0.001 | 0.111 ± 0.002 | 0.088 ± 0.002 | 0.043 ± 0.002 |
| Tyrosine | 0.237 ± 0.003 | 0.130 ± 0.008 | 0.182 ± 0.001 | 0.138 ± 0.007 | 0.177 ± 0.006 | 0.061 ± 0.001 | 0.185 ± 0.002 | 0.138 ± 0.007 | 0.064 ± 0.004 | 0.161 ± 0.004 | 0.071 ± 0.003 | 0.061 ± 0.001 |
| Valine | 0.116 ± 0.006 | 0.073 ± 0.011 | 0.084 ± 0.004 | 0.111 ± 0.010 | 0.063 ± 0.002 | 0.059 ± 0.012 | 0.333 ± 0.004 | 0.218 ± 0.055 | 0.146 ± 0.021 | 0.286 ± 0.172 | 0.095 ± 0.004 | 0.060 ± 0.003 |
| **Organic acids** |  |  |  |  |  |  |  |  |  |  |  |  |
| Ascorbate | 5.509 ± 0.079 | 4.762 ± 0.584 | 4.695 ± 0.711 | 5.036 ± 0.258 | 4.728 ± 0.184 | 3.661 ± 0.186 | 7.227 ± 0.020 | 3.609 ± 0.405 | 1.854 ± 0.625 | 6.167 ± 0.725 | 2.435 ± 0.190 | 1.165 ± 0.041 |
| Citrate | 3.342 ± 0.022 | 7.156 ± 1.709 | 10.456 ± 0.468 | 4.888 ± 0.040 | 6.328 ± 0.501 | 8.461 ± 1.321 | 1.273 ± 0.249 | 1.813 ± 0.126 | 2.310 ± 0.110 | 1.192 ± 0.051 | 1.180 ± 0.050 | 1.557 ± 0.591 |
| Malate | 6.925 ± 0.905 | 15.421 ± 6.764 | 15.610 ± 6.581 | 9.894 ± 0.282 | 13.643 ± 0.692 | 22.019 ± 1.757 | 14.248 ± 2.068 | 9.735 ± 1.265 | 10.137 ± 0.523 | 16.209 ± 0.090 | 10.408 ± 0.321 | 9.006 ± 4.057 |
| Succinate | 0.394 ± 0.004 | 0.241 ± 0.004 | 0.257 ± 0.001 | 0.322 ± 0.001 | 0.282 ± 0.002 | 0.355 ± 0.015 | 0.384 ± 0.003 | 0.324 ± 0.006 | 0.221 ± 0.001 | 0.480 ± 0.001 | 0.330 ± 0.002 | 0.271 ± 0.002 |
| Quinate | 6.347 ± 0.070 | 2.946 ± 1.383 | 3.366 ± 2.618 | 4.543 ± 0.378 | 4.223 ± 0.186 | 2.350 ± 0.025 | 20.211 ± 2.334 | 6.788 ± 3.050 | 1.425 ± 0.123 | 13.831 ± 0.871 | 4.857 ± 0.491 | 0.775 ± 0.351 |
| Lactate | 0.087 ± 0.018 | 0.063 ± 0.003 | 0.054 ± 0.001 | 0.071 ± 0.001 | 0.039 ± 0.001 | 0.026 ± 0.000 | 0.095 ± 0.000 | 0.074 ± 0.001 | 0.072 ± 0.001 | 0.069 ± 0.000 | 0.069 ± 0.003 | 0.069 ± 0.001 |
| Formate | 0.034 ± 0.006 | 0.030 ± 0.001 | 0.031 ± 0.001 | 0.037 ± 0.010 | 0.040 ± 0.002 | 0.035 ± 0.001 | 0.049 ± 0.001 | 0.040 ± 0.002 | 0.030 ± 0.008 | 0.055 ± 0.001 | 0.057 ± 0.004 | 0.044 ± 0.001 |
| Fumarate | 0.042 ± 0.002 | 0.047 ± 0.001 | 0.040 ± 0.000 | 0.080 ± 0.047 | 0.050 ± 0.000 | 0.044 ± 0.002 | 0.121 ± 0.003 | 0.076 ± 0.003 | 0.048 ± 0.007 | 0.196 ± 0.076 | 0.076 ± 0.005 | 0.057 ± 0.032 |
| Pantothenate | 0.056 ± 0.005 | 0.046 ± 0.003 | 0.052 ± 0.004 | 0.040 ± 0.006 | 0.037 ± 0.001 | 0.042 ± 0.013 | 0.084 ± 0.025 | 0.144 ± 0.139 | 0.030 ± 0.001 | 0.106 ± 0.060 | 0.038 ± 0.001 | 0.029 ± 0.000 |
| 3-Hydroxy-3-methylglutarate | 0.275 ± 0.070 | 0.054 ± 0.004 | 0.063 ± 0.002 | 0.093 ± 0.037 | 0.059 ± 0.005 | 0.054 ± 0.001 | 0.759 ± 0.164 | 0.388 ± 0.137 | 0.190 ± 0.071 | 0.228 ± 0.196 | 0.118 ± 0.012 | 0.066 ± 0.004 |

**Table S1.** *Cont.*

| Genotype | **Resolution** | | | **Tora** | | | **Resolution** | | | **Tora** | | |
| --- | --- | --- | --- | --- | --- | --- | --- | --- | --- | --- | --- | --- |
| Tissue | **Leaf** | | | **Leaf** | | | **Stem** | | | **Stem** | | |
| Harvest Position | **Top** | **Middle** | **Bottom** | **Top** | **Middle** | **Bottom** | **Top** | **Middle** | **Bottom** | **Top** | **Middle** | **Bottom** |
| **Organic acids** |  |  |  |  |  |  |  |  |  |  |  |  |
| 3-Hydroxyisovalerate | 0.018 ± 0.002 | 0.027 ± 0.001 | 0.029 ± 0.004 | 0.014 ± 0.002 | 0.024 ± 0.001 | 0.020 ± 0.001 | 0.020 ± 0.001 | 0.014 ± 0.000 | 0.015 ± 0.001 | 0.018 ± 0.001 | 0.013 ± 0.001 | 0.014 ± 0.001 |
| 3-Hydroxymandelate | 0.303 ± 0.009 | 0.385 ± 0.006 | 0.395 ± 0.183 | 0.185 ± 0.007 | 0.262 ± 0.002 | 0.465 ± 0.034 | 0.261 ± 0.025 | 0.205 ± 0.015 | 0.183 ± 0.022 | 0.173 ± 0.020 | 0.160 ± 0.059 | 0.163 ± 0.023 |
| 4-Hydroxy-3-methoxymandelate | 0.333 ± 0.149 | 0.396 ± 0.200 | 0.367 ± 0.058 | 0.365 ± 0.051 | 0.344 ± 0.003 | 0.305 ± 0.036 | 0.192 ± 0.100 | 0.072 ± 0.007 | 0.143 ± 0.073 | 0.127 ± 0.024 | 0.089 ± 0.039 | 0.071 ± 0.013 |
| Acetate | 0.030 ± 0.002 | 0.032 ± 0.002 | 0.028 ± 0.003 | 0.032 ± 0.004 | 0.037 ± 0.001 | 0.040 ± 0.002 | 0.055 ± 0.015 | 0.035 ± 0.018 | 0.033 ± 0.000 | 0.114 ± 0.039 | 0.048 ± 0.003 | 0.038 ± 0.000 |
| 2-Hydroxyisobutyrate | n.d. | 0.024 ± 0.002 | 0.024 ± 0.004 | n.d. | 0.016 ± 0.001 | 0.025 ± 0.003 | n.d. | n.d. | n.d. | n.d. | n.d. | n.d. |
| 2-Oxoglutarate | 1.741 ± 0.184 | 0.983 ± 0.228 | 1.114 ± 0.292 | 1.867 ± 0.084 | 1.439 ± 0.021 | 0.937 ± 0.034 | 6.506 ± 2.081 | 2.194 ± 1.231 | 0.651 ± 0.052 | 6.139 ± 0.066 | 2.367 ± 0.844 | 0.838 ± 0.072 |
| **Aromatics** |  |  |  |  |  |  |  |  |  |  |  |  |
| 2-Phenylethylamine | 1.212 ± 0.011 | 1.282 ± 0.026 | 0.535 ± 0.063 | 0.847 ± 0.043 | 0.558 ± 0.053 | 0.184 ± 0.008 | 2.950 ± 0.221 | 1.645 ± 0.003 | 0.310 ± 0.007 | 3.250 ± 0.111 | 1.205 ± 0.058 | 0.275 ± 0.052 |
| Catechin | 3.438 ± 0.104 | 1.651 ± 0.115 | 1.614 ± 0.221 | 3.913 ± 0.395 | 2.037 ± 0.239 | 1.879 ± 0.053 | 1.702 ± 0.152 | 1.254 ± 0.103 | 1.708 ± 0.037 | 1.535 ± 0.162 | 1.075 ± 0.117 | 1.287 ± 0.356 |
| Chlorogenic Acid | 1.174 ± 0.130 | 1.386 ± 0.147 | 1.392 ± 0.232 | 0.696 ± 0.034 | 0.588 ± 0.089 | 0.913 ± 0.182 | n.d. | n.d. | n.d. | n.d. | n.d. | n.d. |
| Gallocatechin | 2.638 ± 0.242 | 2.109 ± 0.029 | 1.660 ± 0.017 | 3.149 ± 0.018 | 2.407 ± 0.485 | 2.159 ± 0.450 | 3.431 ± 0.048 | 2.625 ± 0.554 | 2.273 ± 0.024 | 3.959 ± 0.374 | 2.211 ± 0.007 | 1.810 ± 0.446 |
| Dihydromyricetin | 1.309 ± 0.094 | 0.068 ± 0.005 | 0.644 ± 0.003 | 7.553 ± 0.317 | 2.062 ± 0.417 | 0.590 ± 0.016 | 0.458 ± 0.003 | 0.235 ± 0.011 | 1.032 ± 0.567 | 1.430 ± 0.082 | 0.324 ± 0.002 | 0.656 ± 0.003 |
| Salicin | 0.980 ± 0.085 | 0.452 ± 0.000 | 0.589 ± 0.000 | 0.591 ± 0.001 | 0.591 ± 0.000 | 0.590 ± 0.000 | 0.460 ± 0.005 | 0.382 ± 0.009 | 0.203 ± 0.002 | 0.240 ± 0.011 | 0.504 ± 0.168 | 0.932 ± 0.149 |
| Triandrin | 0.562 ± 0.000 | 0.437 ± 0.001 | 0.687 ± 0.002 | 0.229 ± 0.000 | 0.461 ± 0.017 | 0.325 ± 0.005 | 0.590 ± 0.046 | 0.417 ± 0.125 | 0.513 ± 0.033 | 0.674 ± 0.038 | 0.470 ± 0.061 | 0.299 ± 0.021 |
| Trigonelline | 0.120 ± 0.006 | 0.042 ± 0.020 | 0.050 ± 0.010 | 0.130 ± 0.003 | 0.041 ± 0.000 | 0.037 ± 0.006 | 0.073 ± 0.018 | 0.048 ± 0.002 | 0.035 ± 0.000 | 0.082 ± 0.002 | 0.028 ± 0.001 | 0.027 ± 0.002 |
| Uridine | 0.068 ± 0.003 | 0.084 ± 0.000 | 0.239 ± 0.003 | 0.211 ± 0.000 | 0.191 ± 0.003 | 0.361 ± 0.001 | 0.515 ± 0.003 | 0.182 ± 0.000 | 0.017 ± 0.000 | 0.575 ± 0.001 | 0.147 ± 0.002 | 0.113 ± 0.001 |
| **Methyl donors** |  |  |  |  |  |  |  |  |  |  |  |  |
| Betaine | 0.244 ± 0.002 | 0.244 ± 0.007 | 0.247 ± 0.027 | 0.226 ± 0.008 | 0.228 ± 0.008 | 0.187 ± 0.029 | 0.215 ± 0.029 | 0.070 ± 0.000 | 0.046 ± 0.003 | 0.157 ± 0.001 | 0.075 ± 0.068 | 0.030 ± 0.001 |
| Choline | 1.136 ± 0.016 | 0.678 ± 0.101 | 0.642 ± 0.184 | 1.086 ± 0.051 | 0.759 ± 0.009 | 0.597 ± 0.032 | 1.109 ± 0.056 | 0.754 ± 0.002 | 0.633 ± 0.004 | 1.300 ± 0.032 | 0.721 ± 0.004 | 0.689 ± 0.046 |
| **Totalⱡ** | 86.892 ± 3.20 | 84.760 ± 5.46 | 86.652 ± 2.49 | 92.778 ± 2.30 | 87.666 ± 2.69 | 75.479 ± 4.26 | 186.568 ± 4.71 | 97.158 ± 15.14 | 66.097 ± 3.28 | 184.753 ± 3.78 | 84.356 ± 4.80 | 52.888 ± 6.78 |

Values represent mean ± standard deviation of two biological replicates. n.d. = not detected.

ⱡ Total metabolite mass in mg/g dry weight represents a sum of all quantified metabolite masses.

© 2014 by the authors; licensee MDPI, Basel, Switzerland. This article is an open access article distributed under the terms and conditions of the Creative Commons Attribution license (http://creativecommons.org/licenses/by/4.0/).
