# Supplementary material for: An Efficient High Throughput Metabotyping Platform for Screening of Biomass Willows
Source: Metabolites. 2014 Oct 28;4(4):946–76. doi: 10.3390/metabo4040946 (PMC4279154; doi:10.3390/metabo4040946)
Supplement: Supplementary File 1 [file metabolites-04-00946-s001.zip › metabolites-64902-sup-update/Table S2.docx]

**Table S2.** Concentrations of 52 metabolites quantified from 1D ^1^H-NMR data of willow leaf and stem tissue *via* Chenomx software against a library of 90 compound signatures run under identical conditions. Values expressed as micrograms per mg of the extractable metabolite pool.

| **Genotype** | **Resolution** | | | **Tora** | | | **Resolution** | | | **Tora** | | |
| --- | --- | --- | --- | --- | --- | --- | --- | --- | --- | --- | --- | --- |
| **Tissue** | **Leaf** | | | **Leaf** | | | **Stem** | | | **Stem** | | |
| **Position** | **T** | **M** | **B** | **T** | **M** | **B** | **T** | **M** | **B** | **T** | **M** | **B** |
| **Carbohydrates** |  |  |  |  |  |  |  |  |  |  |  |  |
| Sucrose | 44.079 ± 0.038 | 34.787 ± 3.305 | 27.962 ± 3.875 | 43.640 ± 2.037 | 41.653 ± 0.357 | 11.983 ± 0.078 | 9.950 ± 0.267 | 25.228 ± 2.695 | 35.280 ± 3.657 | 11.369 ± 0.676 | 35.444 ± 3.068 | 39.246 ± 5.818 |
| Glucose | 45.469 ± 5.419 | 28.065 ± 5.799 | 32.755 ± 17.000 | 35.325 ± 1.266 | 31.912 ± 0.260 | 16.978 ± 0.293 | 93.825 ± 24.357 | 57.891 ± 10.488 | 55.007 ± 13.140 | 117.154 ± 3.002 | 68.028 ± 27.182 | 61.006 ± 19.482 |
| Fructose | 24.335 ± 9.155 | 18.729 ± 5.418 | 23.589 ± 7.673 | 19.225 ± 6.010 | 16.239 ± 0.539 | 11.021 ± 4.186 | 97.402 ± 17.802 | 67.375 ± 16.339 | 41.768 ± 34.684 | 138.985 ± 8.623 | 99.476 ± 26.449 | 91.467 ± 11.422 |
| Maltose | 1.469 ± 0.017 | 1.094 ± 0.102 | 1.382 ± 0.222 | 1.525 ± 0.097 | 1.455 ± 0.007 | 1.169 ± 0.028 | 1.410 ± 0.157 | 1.962 ± 0.237 | 2.342 ± 0.209 | 1.595 ± 0.685 | 1.775 ± 0.124 | 1.852 ± 0.554 |
| Raffinose | 1.799 ± 0.019 | 4.846 ± 3.869 | 2.536 ± 0.579 | 2.518 ± 0.137 | 2.397 ± 0.011 | 2.367 ± 0.117 | 2.094 ± 0.060 | 3.782 ± 0.452 | 5.012 ± 0.460 | 0.663 ± 0.038 | 0.859 ± 0.020 | 2.303 ± 0.931 |
| Stachyose | 2.200 ± 0.041 | 0.978 ± 0.135 | 3.969 ± 0.597 | 1.174 ± 0.119 | 2.705 ± 0.392 | 4.767 ± 0.191 | 2.632 ± 0.101 | 4.737 ± 0.601 | 5.399 ± 0.561 | 0.374 ± 0.021 | 1.537 ± 0.077 | 2.674 ± 0.420 |
| Galactose | 1.049 ± 0.034 | 1.181 ± 0.099 | 1.052 ± 0.464 | 7.978 ± 1.281 | 13.977 ± 8.475 | 2.143 ± 1.450 | 2.038 ± 0.023 | 2.080 ± 0.464 | 3.156 ± 0.588 | 1.832 ± 0.159 | 1.579 ± 0.345 | 1.863 ± 2.635 |
| Fucose | 0.683 ± 0.091 | 0.726 ± 0.334 | 0.717 ± 0.117 | 0.615 ± 0.038 | 0.514 ± 0.032 | 0.410 ± 0.156 | 0.734 ± 0.104 | 0.719 ± 0.160 | 0.904 ± 0.138 | 0.326 ± 0.003 | 0.443 ± 0.186 | 1.091 ± 0.584 |
| **Sugar alcohols** |  |  |  |  |  |  |  |  |  |  |  |  |
| Myo-inositol | 22.009 ± 0.835 | 19.669 ± 0.131 | 22.155 ± 5.912 | 25.131 ± 5.983 | 27.033 ± 1.745 | 21.356 ± 2.290 | 19.877 ± 2.567 | 9.317 ± 0.247 | 10.167 ± 3.919 | 18.395 ± 1.350 | 9.558 ± 0.102 | 3.963 ± 5.605 |
| **Amino acids** |  |  |  |  |  |  |  |  |  |  |  |  |
| Glutamate | 9.158 ± 0.021 | 5.478 ± 0.434 | 5.282 ± 0.781 | 8.034 ± 1.080 | 5.264 ± 0.064 | 3.380 ± 0.218 | 2.732 ± 0.079 | 7.069 ± 0.804 | 10.577 ± 1.080 | 3.859 ± 0.189 | 7.772 ± 0.683 | 11.804 ± 1.738 |
| Glutamine | 7.215 ± 0.208 | 3.127 ± 0.550 | 3.402 ± 0.557 | 7.399 ± 0.303 | 2.026 ± 0.013 | 3.260 ± 0.241 | 42.071 ± 9.316 | 55.155 ± 17.864 | 39.777 ± 12.038 | 24.330 ± 0.155 | 23.087 ± 2.397 | 12.959 ± 0.397 |
| Glycine | 1.909 ± 0.095 | 0.834 ± 0.057 | 1.378 ± 0.148 | 1.485 ± 0.077 | 1.607 ± 0.052 | 1.280 ± 0.026 | 8.480 ± 0.400 | 6.407 ± 0.870 | 1.109 ± 0.048 | 11.809 ± 0.786 | 10.190 ± 0.275 | 7.956 ± 0.709 |
| Alanine | 1.777 ± 0.184 | 0.572 ± 0.212 | 0.613 ± 0.382 | 1.641 ± 0.177 | 0.667 ± 0.135 | 0.224 ± 0.026 | 1.957 ± 0.293 | 1.984 ± 0.301 | 2.324 ± 0.013 | 1.633 ± 0.432 | 1.071 ± 0.014 | 1.323 ± 0.282 |
| Arginine | 1.675 ± 0.014 | 1.086 ± 0.086 | 1.395 ± 0.186 | 1.257 ± 0.054 | 1.255 ± 0.013 | 1.752 ± 0.136 | 2.097 ± 0.077 | 4.307 ± 0.497 | 3.474 ± 0.328 | 1.902 ± 0.103 | 3.049 ± 0.236 | 2.439 ± 0.471 |
| Aspartate | 0.065 ± 0.004 | 0.031 ± 0.006 | 0.060 ± 0.008 | 0.072 ± 0.005 | 0.042 ± 0.000 | 0.065 ± 0.006 | 0.134 ± 0.003 | 0.150 ± 0.030 | 0.146 ± 0.053 | 0.091 ± 0.002 | 0.109 ± 0.009 | 0.101 ± 0.022 |
| Asparagine | 2.570 ± 0.000 | 1.209 ± 0.130 | 0.895 ± 0.140 | 0.994 ± 0.062 | 0.736 ± 0.009 | 0.736 ± 0.041 | 54.030 ± 17.935 | 65.950 ± 24.861 | 60.391 ± 26.298 | 13.756 ± 0.602 | 13.821 ± 0.990 | 8.741 ± 2.674 |
| GABA | 0.416 ± 0.099 | 0.425 ± 0.044 | 0.562 ± 0.058 | 0.323 ± 0.003 | 0.348 ± 0.013 | 0.418 ± 0.001 | 4.857 ± 0.732 | 5.959 ± 0.278 | 3.357 ± 1.646 | 7.716 ± 2.974 | 3.470 ± 0.297 | 3.208 ± 1.253 |
| Isoleucine | 0.445 ± 0.087 | 0.245 ± 0.036 | 0.302 ± 0.062 | 0.282 ± 0.037 | 0.201 ± 0.064 | 0.277 ± 0.241 | 0.839 ± 0.063 | 1.114 ± 0.126 | 0.919 ± 0.132 | 0.607 ± 0.472 | 0.372 ± 0.004 | 0.337 ± 0.046 |
| Lysine | 0.950 ± 0.064 | 0.622 ± 0.027 | 0.579 ± 0.085 | 0.552 ± 0.036 | 0.527 ± 0.004 | 0.912 ± 0.039 | 0.113 ± 0.005 | 0.411 ± 0.056 | 0.975 ± 0.141 | 0.137 ± 0.007 | 0.241 ± 0.018 | 1.391 ± 0.215 |
| Leucine | 0.173 ± 0.009 | 0.129 ± 0.011 | 0.250 ± 0.036 | 0.277 ± 0.015 | 0.190 ± 0.003 | 0.140 ± 0.002 | 0.955 ± 0.033 | 0.953 ± 0.119 | 0.642 ± 0.041 | 0.620 ± 0.051 | 0.459 ± 0.073 | 0.496 ± 0.037 |
| Methionine | 0.181 ± 0.000 | 0.224 ± 0.074 | 0.078 ± 0.012 | 0.259 ± 0.014 | 0.133 ± 0.002 | 0.180 ± 0.011 | 1.197 ± 0.047 | 1.569 ± 0.155 | 1.484 ± 0.207 | 1.146 ± 0.084 | 1.074 ± 0.068 | 0.253 ± 0.046 |
| Threonine | 1.427 ± 0.365 | 0.300 ± 0.109 | 0.393 ± 0.182 | 0.940 ± 0.071 | 0.354 ± 0.011 | 0.164 ± 0.075 | 3.005 ± 0.581 | 2.907 ± 0.838 | 1.866 ± 0.446 | 1.301 ± 0.359 | 0.877 ± 0.040 | 0.809 ± 0.058 |
| Tryptophan | 0.982 ± 0.169 | 0.800 ± 0.117 | 0.893 ± 0.272 | 1.430 ± 0.489 | 1.474 ± 0.175 | 1.333 ± 0.179 | 0.586 ± 0.030 | 0.421 ± 0.083 | 0.307 ± 0.038 | 0.318 ± 0.026 | 0.451 ± 0.023 | 0.341 ± 0.066 |
| Tyrosine | 0.843 ± 0.014 | 0.381 ± 0.060 | 0.574 ± 0.082 | 0.481 ± 0.002 | 0.587 ± 0.024 | 0.185 ± 0.006 | 0.537 ± 0.026 | 0.714 ± 0.051 | 0.437 ± 0.015 | 0.462 ± 0.042 | 0.363 ± 0.014 | 0.487 ± 0.065 |
| Valine | 0.415 ± 0.023 | 0.212 ± 0.050 | 0.265 ± 0.026 | 0.384 ± 0.015 | 0.209 ± 0.004 | 0.180 ± 0.043 | 0.966 ± 0.048 | 1.116 ± 0.152 | 0.999 ± 0.053 | 0.834 ± 0.546 | 0.488 ± 0.018 | 0.474 ± 0.054 |
| **Organic acids** |  |  |  |  |  |  |  |  |  |  |  |  |
| Ascorbate | 19.656 ± 0.141 | 13.853 ± 2.904 | 14.939 ± 4.393 | 17.485 ± 0.050 | 15.719 ± 0.555 | 10.988 ± 0.061 | 20.934 ± 0.721 | 18.595 ± 0.138 | 12.596 ± 3.206 | 17.734 ± 3.210 | 12.443 ± 0.009 | 9.188 ± 1.174 |
| Citrate | 11.919 ± 0.156 | 20.484 ± 3.134 | 32.842 ± 3.392 | 17.027 ± 0.828 | 21.012 ± 1.785 | 25.542 ± 5.137 | 3.692 ± 0.847 | 9.378 ± 0.471 | 15.890 ± 0.649 | 3.410 ± 0.077 | 6.057 ± 0.720 | 12.017 ± 2.766 |
| Malate | 24.682 ± 3.071 | 43.702 ± 15.733 | 47.640 ± 13.489 | 34.493 ± 2.931 | 45.352 ± 2.123 | 66.306 ± 8.287 | 41.098 ± 4.547 | 50.112 ± 0.507 | 69.649 ± 2.640 | 46.415 ± 2.750 | 53.299 ± 2.454 | 69.044 ± 20.988 |
| Succinate | 1.402 ± 0.008 | 0.697 ± 0.071 | 0.810 ± 0.122 | 1.122 ± 0.066 | 0.936 ± 0.002 | 1.067 ± 0.004 | 1.113 ± 0.048 | 1.680 ± 0.169 | 1.515 ± 0.152 | 1.376 ± 0.086 | 1.691 ± 0.120 | 2.151 ± 0.350 |
| Quinate | 22.750 ± 0.035 | 8.544 ± 4.534 | 11.209 ± 9.809 | 15.845 ± 2.188 | 14.039 ± 0.565 | 7.070 ± 0.387 | 58.338 ± 4.702 | 34.270 ± 11.607 | 9.743 ± 0.083 | 39.543 ± 0.093 | 24.808 ± 0.600 | 5.931 ± 1.820 |
| Formate | 0.122 ± 0.021 | 0.085 ± 0.002 | 0.097 ± 0.016 | 0.131 ± 0.043 | 0.133 ± 0.006 | 0.105 ± 0.003 | 0.141 ± 0.008 | 0.210 ± 0.034 | 0.203 ± 0.038 | 0.158 ± 0.007 | 0.292 ± 0.041 | 0.352 ± 0.063 |
| Fumarate | 0.148 ± 0.008 | 0.137 ± 0.015 | 0.126 ± 0.020 | 0.296 ± 0.197 | 0.166 ± 0.000 | 0.131 ± 0.001 | 0.348 ± 0.002 | 0.395 ± 0.030 | 0.331 ± 0.022 | 0.568 ± 0.254 | 0.389 ± 0.058 | 0.430 ± 0.186 |

**Table S2.** *Cont.*

| **Genotype** | **Resolution** | | | **Tora** | | | **Resolution** | | | **Tora** | | |
| --- | --- | --- | --- | --- | --- | --- | --- | --- | --- | --- | --- | --- |
| **Tissue** | **Leaf** | | | **Leaf** | | | **Stem** | | | **Stem** | | |
| **Position** | **T** | **M** | **B** | **T** | **M** | **B** | **T** | **M** | **B** | **T** | **M** | **B** |
| **Organic acids** |  |  |  |  |  |  |  |  |  |  |  |  |
| Pantothenate | 0.199 ± 0.018 | 0.125 ± 0.006 | 0.163 ± 0.009 | 0.140 ± 0.014 | 0.122 ± 0.002 | 0.126 ± 0.033 | 0.245 ± 0.083 | 0.802 ± 0.830 | 0.206 ± 0.015 | 0.308 ± 0.191 | 0.195 ± 0.010 | 0.235 ± 0.038 |
| 3-Hydroxy-3-methylglutarate | 0.980 ± 0.242 | 0.158 ± 0.027 | 0.198 ± 0.024 | 0.330 ± 0.152 | 0.196 ± 0.013 | 0.161 ± 0.010 | 2.185 ± 0.399 | 1.970 ± 0.469 | 1.285 ± 0.373 | 0.673 ± 0.603 | 0.602 ± 0.016 | 0.522 ± 0.052 |
| 3-Hydroxy  isovalerate | 0.066 ± 0.007 | 0.075 ± 0.002 | 0.090 ± 0.025 | 0.050 ± 0.011 | 0.080 ± 0.003 | 0.061 ± 0.000 | 0.057 ± 0.002 | 0.072 ± 0.011 | 0.100 ± 0.001 | 0.051 ± 0.000 | 0.067 ± 0.010 | 0.115 ± 0.013 |
| 3-Hydroxy  mandelate | 1.107 ± 0.077 | 1.101 ± 0.064 | 1.198 ± 0.401 | 0.643 ± 0.010 | 0.871 ± 0.002 | 1.396 ± 0.039 | 0.755 ± 0.042 | 1.066 ± 0.207 | 1.291 ± 0.237 | 0.493 ± 0.028 | 0.812 ± 0.241 | 1.288 ± 0.029 |
| 4-Hydroxy-3-methoxy  mandelate | 1.198 ± 0.527 | 1.086 ± 0.528 | 1.142 ± 0.027 | 1.271 ± 0.245 | 1.142 ± 0.004 | 0.918 ± 0.150 | 0.550 ± 0.274 | 0.375 ± 0.079 | 0.976 ± 0.442 | 0.361 ± 0.086 | 0.452 ± 0.170 | 0.558 ± 0.014 |
| Acetate | 0.109 ± 0.009 | 0.093 ± 0.014 | 0.090 ± 0.023 | 0.111 ± 0.019 | 0.124 ± 0.001 | 0.119 ± 0.001 | 0.159 ± 0.048 | 0.185 ± 0.117 | 0.227 ± 0.018 | 0.329 ± 0.133 | 0.247 ± 0.037 | 0.299 ± 0.048 |
| 2-Hydroxy  isobutyrate | n.d. | 0.070 ± 0.012 | 0.076 ± 0.002 | n.d. | 0.054 ± 0.003 | 0.074 ± 0.012 | n.d. | n.d. | n.d. | n.d. | n.d. | n.d. |
| 2_Oxoglutarate | 6.248 ± 0.561 | 2.786 ± 0.787 | 3.561 ± 1.409 | 6.520 ± 0.668 | 4.770 ± 0.109 | 2.811 ± 0.022 | 18.783 ± 5.265 | 10.994 ± 5.022 | 4.449 ± 0.029 | 17.575 ± 0.964 | 11.958 ± 3.411 | 6.626 ± 0.481 |
| **Aromatics** |  |  |  |  |  |  |  |  |  |  |  |  |
| 2-Phenyl-ethylamine | 4.320 ± 0.015 | 3.689 ± 0.226 | 1.668 ± 0.049 | 2.946 ± 0.016 | 1.856 ± 0.182 | 0.550 ± 0.002 | 8.526 ± 0.911 | 8.539 ± 1.035 | 2.150 ± 0.249 | 9.296 ± 0.278 | 6.191 ± 0.771 | 2.221 ± 0.752 |
| Catechin | 12.260 ± 0.451 | 4.759 ± 0.084 | 5.029 ± 0.054 | 13.588 ± 0.605 | 6.780 ± 0.812 | 5.650 ± 0.414 | 4.912 ± 0.270 | 6.540 ± 1.312 | 11.777 ± 1.321 | 4.375 ± 0.182 | 5.535 ± 1.029 | 10.011 ± 1.225 |
| Chlorogenic Acid | 4.190 ± 0.495 | 4.058 ± 0.818 | 4.325 ± 0.092 | 2.424 ± 0.020 | 1.954 ± 0.303 | 2.756 ± 0.671 | n.d. | n.d. | n.d. | n.d. | n.d. | n.d. |
| Gallocatechin | 9.394 ± 0.890 | 6.049 ± 0.545 | 5.217 ± 0.802 | 10.996 ± 0.711 | 7.981 ± 1.679 | 6.509 ± 1.630 | 9.905 ± 0.460 | 13.791 ± 4.514 | 15.611 ± 1.237 | 11.290 ± 0.328 | 11.319 ± 0.909 | 14.068 ± 1.309 |
| Dihydromyricetin | 4.662 ± 0.311 | 0.200 ± 0.036 | 2.030 ± 0.311 | 26.279 ± 0.385 | 6.855 ± 1.414 | 1.775 ± 0.127 | 1.323 ± 0.051 | 1.223 ± 0.203 | 7.267 ± 4.549 | 4.087 ± 0.031 | 1.660 ± 0.116 | 5.211 ± 0.815 |
| Salicin | 3.473 ± 0.295 | 1.282 ± 0.079 | 1.855 ± 0.272 | 2.057 ± 0.111 | 1.962 ± 0.010 | 1.775 ± 0.080 | 1.329 ± 0.030 | 1.985 ± 0.287 | 1.395 ± 0.114 | 0.686 ± 0.014 | 2.563 ± 0.650 | 7.437 ± 0.141 |
| Triandrin | 2.004 ± 0.014 | 1.267 ± 0.115 | 2.162 ± 0.322 | 0.797 ± 0.044 | 1.531 ± 0.063 | 0.976 ± 0.030 | 1.704 ± 0.072 | 2.122 ± 0.391 | 3.553 ± 0.535 | 1.933 ± 0.235 | 2.394 ± 0.130 | 2.375 ± 0.548 |
| Trigonelline | 0.429 ± 0.023 | 0.120 ± 0.062 | 0.158 ± 0.054 | 0.451 ± 0.016 | 0.137 ± 0.001 | 0.113 ± 0.024 | 0.209 ± 0.053 | 0.247 ± 0.017 | 0.239 ± 0.016 | 0.235 ± 0.019 | 0.144 ± 0.015 | 0.216 ± 0.045 |
| Uridine | 0.243 ± 0.008 | 0.243 ± 0.020 | 0.753 ± 0.119 | 0.735 ± 0.040 | 0.634 ± 0.012 | 1.085 ± 0.046 | 1.489 ± 0.045 | 0.946 ± 0.113 | 0.120 ± 0.011 | 1.647 ± 0.110 | 0.755 ± 0.048 | 0.901 ± 0.130 |
| **Methyl donors** |  |  |  |  |  |  |  |  |  |  |  |  |
| Betaine | 0.870 ± 0.003 | 0.708 ± 0.045 | 0.782 ± 0.197 | 0.785 ± 0.015 | 0.756 ± 0.030 | 0.563 ± 0.111 | 0.626 ± 0.057 | 0.366 ± 0.043 | 0.316 ± 0.005 | 0.450 ± 0.026 | 0.374 ± 0.319 | 0.240 ± 0.033 |
| Choline | 4.048 ± 0.035 | 1.973 ± 0.462 | 2.064 ± 0.878 | 3.779 ± 0.035 | 2.524 ± 0.022 | 1.796 ± 0.178 | 3.204 ± 0.052 | 3.911 ± 0.477 | 4.363 ± 0.411 | 3.719 ± 0.149 | 3.699 ± 0.309 | 5.453 ± 0.484 |
| **Total^ⱡ^** | 309.77 ± 24.34 | 243.10 ±51.52 | 273.26 ±75.76 | 323.24 ± 28.83 | 291.19 ± 22.11 | 227.10 ±27.54 | 527.92 ±31.24 | 433.54 ±74.90 | 415.47 ± 89.09 | 536.14 ± 94.09 | 499.05 ±106.85 | 453.07 ±116.29 |

Values represent mean ± standard deviation of two biological replicates. n.d. = not detected.

ⱡ Total metabolite mass represents a sum of all quantified metabolite masses.

© 2014 by the authors; licensee MDPI, Basel, Switzerland. This article is an open access article distributed under the terms and conditions of the Creative Commons Attribution license (http://creativecommons.org/licenses/by/4.0/).
