# Supplementary material for: An Efficient High Throughput Metabotyping Platform for Screening of Biomass Willows
Source: Metabolites. 2014 Oct 28;4(4):946–76. doi: 10.3390/metabo4040946 (PMC4279154; doi:10.3390/metabo4040946)
Supplement: Supplementary File 1 [file metabolites-04-00946-s001.zip › metabolites-64902-sup-update/Table S3.docx]

**Table S3.** Characteristic 1D ^1^H-NMR chemical shift regions (ppm) of 52 metabolites found in willow leaf and stem tissue, extracted in 80:20 D_2_O:CD_3_OD (plus EDTA and buffered to pH 7.4). Chemical shift values are relative to d_4_-TSP and have been obtained directly from the Chenomx library of signatures of authentic standards run under identical conditions. In Chenomx all peaks, for each metabolite, are used for matching and quantitation.

| **Metabolite** | **Characteristic Chemical Shift Assignments (ppm)** |
| --- | --- |
| Pantothenate | 0.899 (s, 6-H_3_/7-H_3_); 0.931 (s, 6-H_3_/7-H_3_) |
| Isoleucine | 0.930 (t, *J* = 7.35 Hz, 5-H_3_); 0.981 (d, *J* = 7Hz, 6-H_3_) |
| Isoleucine | 0.953 ( 2 x d, J *=* 7 Hz, 5-H_3_ and 6-H_3)_ |
| Valine | 0.981 (d, *J* =7.0 Hz, 4-H_3_); 1.033 (d, *J* = 7.05 Hz, 5-H_3_) |
| α-Fucose  β-Fucose | 1.211 (d, *J* = 6.56 Hz, 6-H_3_); 5.195 (d, *J* = 3.65 Hz, 1-H)  1.236 (d, *J* = 6.46 Hz, 6-H_3_); 4.55 (d, *J* = 7.84 Hz, 1-H) |
| 3-Hydroxyisovalerate | 1.256 (s, 4-H_3_ and 5-H_3_) |
| 3-Hydroxy-3-methylglutarate | 1.307 (s, 6-H_3_) |
| Lactate | 1.312 (d, *J* = 6.73 Hz, 3-H_3_) |
| Threonine | 1.316 (d, *J* = 6.44 Hz, 4-H_3_) |
| 2-Hydroxyisobutyrate | 1.339 (s, 3-H_3_ and 4-H_3_) |
| Alanine | 1.466 (d, *J* = 7.25 Hz, 3-H_3_) |
| Quinate | 1.861 (dd, *J* = 10.38, 13.2 Hz, 3-H)  1.933 (ddd, *J* = 2.2, 3.59, 14.2 Hz, 7-H)  2.027 (m, 3-H and 7-H) |
| Acetate | 1.896 (s, 2-H_3_) |
| Chlorogenic acid | 2.00 (m, 3-H and 7-H of quinate moiety)  2.126 (m, 3-H and 7-H of quinate moiety)  5.31 (m, 6-H of quinate moiety)  6.380 (d, *J* = 15.35 Hz, 8-H of caffeoyl moiety)  6.902 (d, *J* = 8 Hz, 3-H of caffeoyl aromatic ring)  7.907 (dd, *J* = 2, 8 Hz, 4-H of caffeoyl aromatic ring)  7.173 (d, *J* = 2 Hz, 6-H of caffeoyl aromatic ring)  7.641 (d, *J* = 15.7 Hz, 7-H of caffeoyl moiety) |
| Glutamate | 2.049 (m, 3-H); 2.108 (m, 3-H); 2.389 (m, 4-H_2_); 3.719 (dd, J = 4.95, 7 Hz, 2-H) |
| Glutamine | 2.113 (m, 3-H_2_); 2.435 (m, 4-H_2_), 3.724 (t, *J* = 6.1 Hz, 2-H) |
| Malate | 2.334 (dd, *J* = 10, 15.2 Hz, 3-H); 2.648 (dd, *J* = 2.9, 15.1 Hz, 3-H); 4.269 (dd, *J* = 2.8, 10 Hz, 2-H) |
| Succinate | 2.384 (s, 2-H_2_ and 3-H_2_) |
| 2-Oxoglutarate | 2.425 (t, *J* = 6.6 Hz, 4-H_2_); 2.981 (t, *J* = 6.87 Hz, 3-H_2_) |
| Citrate | 2.52 (d, *J* = 15.2 Hz); 2.655 (d, *J* = 15.2 Hz) |
| Catechin | 2.848 (dd, *J* = 5.3, 15.8 Hz, 7-H); 4.762 ( d, *J* = 7 Hz, 9-H); 6.835 (dd, *J* = 2, 8 Hz, 15-H); 6.908 (d, *J* = 8 Hz, 14-H); 6.918 (d, *J* = 2 Hz, 11-H) |
| 2-Phenylethylamine | 2.986 (t, *J* = 7.29 Hz, 2-H_2_); 3.249 (t, *J* = 7.3 Hz, 1-H_2_); 7.325 -7.405 (m,5 x Ar-H) |
| Choline | 3.181 (s) –N^+^-(CH_3_)_3_ |
| Glycine Betaine | 3.248 (s), –N^+^-(CH_3_)_3_ |
| *Myo*-inositol | 3.248 (t, *J* =- 9.3 Hz, 2-H); 3.497 (dd, *J* = 2.9, 10Hz, 6-H and 4-H); 3.606 (t, *J* = 9.6Hz, 1-H and 3-H); 4.03 (t, *J* = 2.5 Hz, 5-H) |
| Stachyose | 5.413 (d, *J* = 3.8 Hz, 7-H); 4.973 (t, *J* = 3.75 Hz, 13-H and 19-H) |
| Raffinose | 5.409 (d, *J* = 3.5 Hz, 7-H); 4.967 (d, *J* = 3.75 Hz, 13-H) |

**Table S3.** *Cont.*

| **Metabolite** | **Characteristic Chemical Shift Assignments (ppm)** |
| --- | --- |
| Sucrose | 5.396 (d, *J* = 3.8 Hz, 7-H); 4.187 (d, *J* = 8.5 Hz, 2-H); 4.028 (t, *J* = 8.45 Hz, 3-H); 3.741 (t, *J* = 9.2 Hz 9-H); 3.656 (s, 6-H_2_), 3.526 (dd, *J* = 3.65, 9.8 Hz, 8-H); 3.443 (t, *J* = 9.3 Hz, 10-H) |
| Glucose | 5.200 (d, *J* = 3.6, 1-H – alpha glucose); 4.609 (d, *J* = 7.8 Hz, 1-H – beta glucose); 3.213 (dd, *J* = 7.95, 9.4 Hz, 2-H) |
| 4-Hydroxy-3-methoxymandelate | 3.873 (s, OCH_3_); 4.92 (s, 7-H) |
| Fructose | 4.082 (m); 4.012 (dd, *J* = 1.18, 12.65 Hz, 6-H); 3.963 (m, 4-H); 3.866 (dd, *J* = 3.4, 10 Hz, 3-H); |
| Triandrin | 7.390 (d, *J* = 8.6 Hz, 2xAr-H); 6.843 (d, *J* = 8.5 Hz, 2xAr-H); 6.664 (d, *J* = 15.7 Hz, 7-H); 6.234 (dt, *J* = 6.6, 15.7 Hz, 8-H); 4.501 (d, *J* = 7.85 Hz, 1’-H) |
| Formate | 8.444 (s, 1-H) |
| Tryptophan | 7.72 (d, *J* = 7.9 Hz, 7-H); 7.505 (d, *J* = 8.2 Hz, 6-H); 7.278 (t, *J* = 0.49 Hz, 2-H); 7.25 (m, 9-H); 7.168 (m, 8-H) |
| Phenylalanine | 7.405 (m, 3-H and 5-H); 7.35 (m, 4-H); 7.32 (m, 2-H and 6-H) |
| 3-Hydroxymandelate | 4.920 (s, 7-H); 7.289 (t, *J* = 7.8 Hz, 3-H); 6.982 (dt, *J* = 1.26, 7.23 Hz, 4-H); 6.900 (m, 6-H); 6.855 (dd, *J* =0.91, 2.53 Hz, 2-H) |
| Asparagine | 2.92 (dd, *J* = 4, 16.5 Hz, 3-H); 2.810 (dd, *J* =7.7, 16.5 Hz, 3-H); 3.943 (dd, *J* = 4.15, 8 Hz, 2-H) |
| Aspartate | 2.848 (dd, *J* = 5.3, 16 Hz, 3-H); 2.726 (dd, *J* = 8, 16 Hz, 3-H); 3.893 (dd, *J* = 3.8, 8.4 Hz, 2-H) |
| Glycine | 3.516 (s, 2-H_2_) |
| GABA | 1.884 (quintet, *J* = 7.4 Hz, 3-H_2_); 2.276 (t, *J* = 7.2 Hz, 2-H_2_); 2.994 (t, *J* = 7.40 Hz, 4-H_2_) |
| Gallocatechin | 6.511 (s, 11-H and 15-H), 4.717 (d, *J* = 7.14 Hz); 3.823 (dd, *J* =4.75, 15.5 Hz) |
| Salicin | 7.397 (dd, *J* = 1.4, 7.4 Hz, 5-H), 7.365 (dt, *J* = 1.75, 8 Hz, 3-H); 7.206 (d, *J* = 8.1 Hz, 2-H); 7.143 (t, *J* = 7.4 Hz, 4-H); 5.081 (d, *J* = 6.6 Hz, 1’-H); 4.73 (d, *J* = 12.4 Hz, 6’-H); 4.674 (d, *J* = 12.4 Hz, 6’-H) |
| Dihydromyricetin | 6.50 (s, 11-H and and 15-H); 4.916 (d, *J* = 10.8 Hz, 8-H); 4.623 (d, *J* = 10.8 Hz, 9-H) |
| Galactose | 5.233 (d, *J* = 3.8 Hz, 1-H (alpha)); 4.539 (d, *J* = 7.7 Hz, 1-H (beta) |
| Fumarate | 6.497 (s, 2-H and 3-H) |
| Trigonelline | 9.115 (m, 1-H); 8.832 (m, 3-H and 5-H); 8.074 (m, 4-H); 4.428 (s, 9-H_3_) |
| Maltose | 5.352 (d, *J* = 3.65, 1-H); 5.189 (d, *J* = 3.65, 10-H) |
| Arginine | 1.642 (m, 4-H); 1.720 (m, 4-H); 1.90 (m, 3-H_2_); 3.225 (t, *J* = 6.85 Hz, 5-H_2_); 3.725 (t, *J* = 6 Hz, 2-H) |
| Lysine | 3.008 (t, *J* = 7.5 Hz, 6-H_2_) |
| Methionine | 2.122 (s, S-CH_3_) |
| Tyrosine | 7.175 (d, *J* = 8 Hz, 2 x Ar-H); 6.868 (d, *J* = 8.3 Hz, 2xAr-H) |
| Ascorbate | 4.467 (d, *J* = 2 Hz, 4-H); 3098 (m, 5-H); 3.714 (m, 6-H_2_) |
| Uridine | 7.87 (d, *J* = 8 Hz, 6-H); 5.89 (*J=* 4.6) and 5.87 ( *J* = 7.8); 4.308 (t, *J* = 4.75 Hz, 2’-H) |

© 2014 by the authors; licensee MDPI, Basel, Switzerland. This article is an open access article distributed under the terms and conditions of the Creative Commons Attribution license (http://creativecommons.org/licenses/by/4.0/).
